# Observation of Excitonic Instability in a Monolayer Ta_2_NiSe_5_ With Strain Disorder

**DOI:** 10.1002/advs.75569

**Published:** 2026-05-11

**Authors:** So Young Kim, Kwangrae Kim, Dowook Kim, Ji Eun Lee, Jieun Seok, Chang Il Kwon, Jo Hyun Yun, Chang‐Jong Kang, Jae Hoon Kim, H. W. Yeom, Tae‐Hwan Kim, Jonghwan Kim, B. J. Kim, Jun Sung Kim

**Affiliations:** ^1^ Department of Physics Pohang University of Science and Technology (POSTECH) Pohang South Korea; ^2^ Department of Materials Science and Engineering Pohang University of Science and Technology Pohang South Korea; ^3^ Department of Physics Yonsei University Seoul South Korea; ^4^ Center for Artificial Low Dimensional Electronic Systems Institute for Basic Science (IBS) Pohang South Korea; ^5^ Department of Physics Chungnam National University Daejeon South Korea; ^6^ Institute for Sciences of the Universe Chungnam National University Daejeon South Korea; ^7^ Center for Van der Waals Quantum Solids Institute for Basic Science (IBS) Pohang South Korea

**Keywords:** exciton‐phonon coupling, excitonic insulating phase, ${\mathrm{Ta}}_{2}$ ${\mathrm{NiSe}}_{5}$

## Abstract

Excitonic insulating phase, a long‐sought exotic quantum state, is formed by spontaneous condensation of electron‐hole pairs or excitons. Stabilizing such a fragile many‐body state in reduced dimensions requires precise control of multiple electronic parameters and external perturbations, which makes its material realization challenging, particularly at high temperatures. Here, using van der Waals layers of ${\mathrm{Ta}}_{2}$ ${\mathrm{NiSe}}_{5}$ as a material platform, we show that the excitonic insulating phase is stable up to $T_{c}$ ∼ 190 K in the two‐dimensional limit. The distinctive signatures of the excitonic insulating phase transition, such as the hybridization gap opening and critical fluctuations of the excitonic order observed in Raman spectroscopy, persist even in a monolayer, although systematically suppressed with thickness. The large gap ratio, nearly independent of thickness, suggests the importance of strong exciton‐phonon coupling in maintaining a high‐temperature excitonic instability, while substrate‐induced strain disorder, as observed by scanning tunneling microscopy, lowers $T_{c}$ in monolayer ${\mathrm{Ta}}_{2}$ ${\mathrm{NiSe}}_{5}$ than in the bulk. Our findings establish the monolayer ${\mathrm{Ta}}_{2}$ ${\mathrm{NiSe}}_{5}$ as a promising model system for studying and manipulating correlated exciton‐lattice coupling at the two dimensional limit.

## Graphical abstract

Monolayer ${\mathrm{Ta}}_{2}$ ${\mathrm{NiSe}}_{5}$ sustains an excitonic insulating phase up to 190 K, with Raman signatures of gap opening and critical excitonic fluctuations. Thickness‐independent large gap ratios highlight strong exciton‐phonon coupling, while substrate‐induced strain disorder depresses *T*_c_, establishing monolayer ${\mathrm{Ta}}_{2}$ ${\mathrm{NiSe}}_{5}$ as a platform for correlated exciton‐lattice physics.

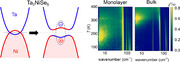

## 1 Introduction

Excitons in two‐dimensional (2D) semiconductors, derived from van der Waals (vdW) materials, exhibit unprecedented optoelectric properties mainly due to the strong Coulomb interaction of space‐confined electron and hole pairs [1, 2, 3]. Strong tunability of exciton's properties with layer thickness [4], strain [5, 6], electric gating [7, 8], and proximity coupling [9, 10, 11, 12] in heterostructures offers a novel route for exploiting excitons and their coupling to other quasi‐particles in the 2D limit. When the excitons' binding energy exceeds the band gap, they can spontaneously condense into a collective state, called an excitonic insulating phase [13, 14, 15, 16]. Realization of such an exotic quantum state may lead to the discovery of novel phenomena and functionalities, including light‐induced phase‐switching of excitonic order, ultra‐sensitive photodetection, and even high‐temperature superconductivity [17, 18, 19]. However, its realization in 2D materials is extremely challenging, particularly at high temperatures. This is because the necessary fine‐tuning of various electronic parameters related to numbers, screening strength, and spatial separation of electrons and holes is difficult to achieve [3] due to their high susceptibility to external perturbations in 2D materials.

An alternative approach is to utilize bulk excitonic insulator candidates and thin them down to monolayer thickness. Among various candidates in van der Waals structure, ${\mathrm{Ta}}_{2}$ ${\mathrm{NiSe}}_{5}$ is one of the leading material platforms for studying excitonic insulating instability, even though the exact nature of its low‐temperature insulating phase still remains under active debate. ${\mathrm{Ta}}_{2}$ ${\mathrm{NiSe}}_{5}$ is a narrow‐gap semimetal or a zero‐gap semiconductor above $T_{c}∼$ 327 K that undergoes a coupled electronic–structural transition into a gapped state, accompanied by the orthorhombic‐to‐monoclinic structural transition [20, 21, 22]. The vital role of the interband Coulomb interaction as the primary driving force, rather than a purely structural mechanism, has been strongly supported by growing evidence, including a divergent excitonic susceptibility, hybridization between a collective phase mode and phonons, collapse of the electronic gap even when the monoclinic distortion persists in a photoinduced nonequilibrium state, and a persistence of a gap in locally orthorhombic regions at structural domain boundaries [22, 23, 24, 25, 26, 27, 28, 29, 30].

Furthermore, several observations in ${\mathrm{Ta}}_{2}$ ${\mathrm{NiSe}}_{5}$, such as its large gap‐to‐$T_{c}$ ratio [26, 31, 32], non‐metallic resistivity with a finite activation gap above $T_{c}$ [33], and clear evidence for preformed excitons above $T_{c}$ [25, 34], place ${\mathrm{Ta}}_{2}$ ${\mathrm{NiSe}}_{5}$ in the strong‐coupling regime. Consistent with this, the spatial extent of the exciton is ∼0.6 nm perpendicular to the chains [25], indicating strong confinement of excitons within the vdW layers and a correspondingly weak thickness dependence of the exciton binding energy. These intriguing features suggest that ${\mathrm{Ta}}_{2}$ ${\mathrm{NiSe}}_{5}$ is a model system for identifying the key parameters for determining the nature of excitonic phase transition at the monolayer limit, where an intrinsic excitonic instability is cooperative with structural instabilities. Here we show that the excitonic instability is indeed present in the monolayer ${\mathrm{Ta}}_{2}$ ${\mathrm{NiSe}}_{5}$ up to $T_{c}∼$ 190 K, somewhat lower than its bulk $T_{c}$, while maintaining its large gap ratio within the strong coupling regime. We identified that in the monolayer limit, the effect of strain disorder, detrimental for high $T_{c}$, dominates over that of enhanced Coulomb interaction by suppressed dielectric screening, highlighting the importance of strain control in the coupled exciton‐lattice instability of ${\mathrm{Ta}}_{2}$ ${\mathrm{NiSe}}_{5}$.

## 2 Results

### 2.1 Raman Spectroscopy on Atomically‐Thin ${\mathrm{Ta}}_{2}$ ${\mathrm{NiSe}}_{5}$ Crystals

**FIGURE 1:**
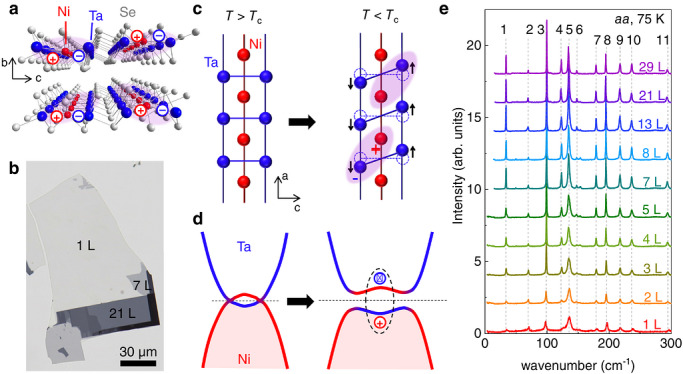
Exciton condensation in van der Waals layered ${\mathrm{Ta}}_{2}$ ${\mathrm{NiSe}}_{5}$. (a) Crystal structure of ${\mathrm{Ta}}_{2}$ ${\mathrm{NiSe}}_{5}$. Electrons and holes at the Ta chains and Ni chains along the a‐axis form layer‐confined excitons. (b) Optical microscope image of mechanically exfoliated ${\mathrm{Ta}}_{2}$ ${\mathrm{NiSe}}_{5}$ monolayer. (c) Schematic illustration of lattice distortion when phase transition occurs. Ta chains shear distort against the Ni chains along the a‐axis. (d) Schematic illustration of band hybridization and gap opening as excitons formed between electrons in the Ta band and holes in the Ni band condensate. (e) Raman spectra in the aa configuration of mechanically exfoliated ${\mathrm{Ta}}_{2}$ ${\mathrm{NiSe}}_{5}$ with different number of layers at 75 K. The dashed lines indicate the eleven phonon modes of ${\mathrm{Ta}}_{2}$ ${\mathrm{NiSe}}_{5}$ single crystal. All spectra are shifted for clarity.

To unveil the excitonic instability in monolayer ${\mathrm{Ta}}_{2}$ ${\mathrm{NiSe}}_{5}$, we utilized polarization‐resolved Raman spectroscopy, a widely‐employed technique for investigating electronic and phononic excitations in atomically thin crystals [35, 36, 37]. In particular, its polarization dependence provides direct insight into the symmetry of excitations, which has been effectively employed to study the nature of the excitonic instability in bulk ${\mathrm{Ta}}_{2}$ ${\mathrm{NiSe}}_{5}$ [22, 26]. In bulk ${\mathrm{Ta}}_{2}$ ${\mathrm{NiSe}}_{5}$, a spontaneous gap opening with a characteristic flattening of the low energy states, consisting of Ta 5d‐derived conduction bands and Se 4p‐Ni 3d‐derived valence bands occurs at $T_{c}$ ∼ 328 K [23, 31, 38, 39], accompanied by the structural phase transition from high‐temperature orthorhombic (Cmcm) to low‐temperature monoclinic (C2/c) structures (Figure 1c) [20]. The exciton formation, which hybridizes the conduction and valence bands belonging to different irreducible representations (IRs) in the orthorhombic structure (Figure 1d), is intimately coupled to the mirror symmetry breaking and $B_{\text{2g}}$ atomic displacements into the monoclinic phase. The resulting excitonic order and its critical fluctuations are manifested by the electronic Raman signatures, i.e. hybridization gap opening at high energies and significant quasi‐elastic scattering at low energies, while the substantial damping and Fano‐line shape of $B_{\text{2g}}$ phonon modes reveal strong exciton‐phonon coupling [22, 26, 29, 40]. The electronic continuum at low energies originates from the strongly damped preformed excitons of the normal semimetallic state above $T_{c}$ and the soft order parameter excitations below $T_{c}$. These characteristic features in the Raman spectra are well preserved but systematically modified with decreasing layer thickness toward the monolayer in ${\mathrm{Ta}}_{2}$ ${\mathrm{NiSe}}_{5}$, as discussed below.

For isolation of monolayer ${\mathrm{Ta}}_{2}$ ${\mathrm{NiSe}}_{5}$ from bulk crystals, we employed the ${\mathrm{Al}}_{2}$ $O_{3}$‐assisted exfoliation method (Figure S1) [41], which utilizes the strong affinity between the contact layers of a vdW crystal and the evaporated ${\mathrm{Al}}_{2}$ $O_{3}$ layers, and successfully obtained monolayer and few‐layer‐thick ${\mathrm{Ta}}_{2}$ ${\mathrm{NiSe}}_{5}$ crystals on top of a sapphire substrate as shown in the optical transmission image of a typical sample (Figure 1b and Figure S2a). The entire processes, including exfoliation, thickness determination, and Raman spectroscopy measurements, were carried out in an inert gas environment or high vacuum, without any exposure to the air (see Experimental Section). For all samples, eleven Raman modes were clearly resolved at 75 K (Figure 1e), as found in the bulk crystal [22, 40]. With x∥a, y∥b, and z∥c, aa and ac polarization configurations correspond to $\bar{y}\left(xx\right)y$ and $\bar{y}\left(xz\right)y$, respectively, in Porto's notation $k_{i}\left(e_{i}e_{s}\right)k_{s}$, where $k_{i}\left(k_{s}\right)$ denotes the propagation direction of the incident (scattered) light and $e_{i}\left(e_{s}\right)$ denotes the polarization of the incident (scattered) light. Furthermore, the consistent optical spectra (Figure S8) indicate that the electronic structure remains essentially unchanged with varying thickness. The M‐shaped dispersion in the electronic structure, which indicates the non‐interacting band structure of a band‐overlap semimetal, also remains essentially unchanged with varying thickness (Figure S6). Thickness‐dependent Raman frequencies taken at 75 K exhibit a systematic blue‐shift with lowering thickness for all modes, except the mode 3 that shows the opposite behavior (Figure S10). Similar blue‐shift of in‐plane Raman modes with lowering thickness has been observed in other 2D semiconductors like ${\mathrm{MoS}}_{2}$ [42], due to suppression of dielectric screening of the long‐range Coulomb interaction with lowering thickness [43]. These observations demonstrate that ultrathin layers of ${\mathrm{Ta}}_{2}$ ${\mathrm{NiSe}}_{5}$ preserve their intrinsic lattice structure without significant degradation during the exfoliation.

**FIGURE 2:**
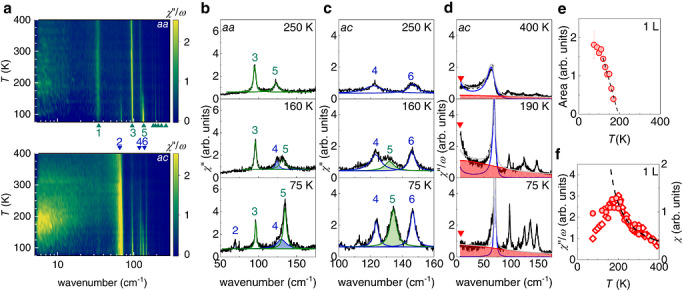
Phase transition even in monolayer ${\mathrm{Ta}}_{2}$ ${\mathrm{NiSe}}_{5}$. (a) False color map of polarized Raman conductivity $\chi^{\prime \prime }$/ω of ${\mathrm{Ta}}_{2}$ ${\mathrm{NiSe}}_{5}$ monolayer from 75 K to 400 K. At 400 K, eight $A_{g}$ modes and three $B_{\text{2g}}$ modes appear isolated in the aa and ac configurations, respectively. (b, c) Raman spectra below (bottom), near (middle), and above (top) the transition temperature in the aa (b) and ac (c) configuration. The green and blue lines represent Lorentzian line‐shaped fit curves for the $A_{g}$ and $B_{\text{2g}}$ phonon modes, respectively. (d) Raman conductivity below (bottom), near (middle), and above (top) transition temperature in the ac configuration. Blue and red curves are fits to the phononic and electronic parts, respectively, and the grey curve shows the total contribution. The area of the red‐shaded region returns the real part of the uniform static susceptibility. (e) Integrated area of the phonon mode 5 as a function of temperature. (f) Raman conductivity at 7 ${\mathrm{cm}}^{-1}$ (diamond) and the static susceptibility (circle) following Curie‐Weiss behavior (dashed curve) above the transition temperature.

### 2.2 Excitonic Instability in the Monolayer

Clear hallmarks of the phase transition in a monolayer ${\mathrm{Ta}}_{2}$ ${\mathrm{NiSe}}_{5}$ are observed in the temperature‐dependent Raman spectroscopy. The Raman conductivity $\chi^{\prime \prime }\left(\omega ,T\right)/\omega$, where $\chi^{\prime \prime }$ is the Bose‐factor‐corrected Raman response obtained from the measured intensity [26], and ω is the Raman shift, for both aa and ac configurations (Figure 2a) reveals 11 modes at low temperatures, all of which are the $A_{g}$ modes in the low‐T monoclinic (C2/c) structure, in good agreement with the bulk case [22, 26, 29, 40, 44]. Upon increasing temperature, the modes 2 and 4, clearly discernible at low temperatures in the aa configuration, become weaker and eventually disappear at $T_{c}$ ∼ 190 K as shown for three representative temperatures across $T_{c}$ in Figure 2b. Consistently, in the ac configuration, the mode 5 seen at low temperatures disappears across $T_{c}$ (Figure 2c,e). From the intensity variation with the azimuthal angle of the incident light polarization against the a‐axis (Figure S9), we found that these modes indeed belong to different IRs, $B_{\text{2g}}$ and $A_{g}$ in the high‐T orthorhombic (Cmcm) structure. These observations resemble the bulk case, in which the 11 Raman active modes at low temperatures branch into three $B_{\text{2g}}$ modes in the ac configuration and eight $A_{g}$ modes in the aa configuration [22, 26, 29, 40, 44] due to the structural transition. We take the phonon mode 5 in the ac configuration and track its intensity as a function of temperature by fitting the Raman spectra with a Lorentzian line shape. This intensity abruptly drops to zero at $T_{c}∼$ 190 K, somewhat lower than $T_{c}$ ∼ 327 K of the bulk sample (Figure 2e and Figures S13a and S15). The selective disappearance of the Raman modes, the same as the bulk case, confirms that the same structural transition occurs even in the monolayer.

The electronic Raman scattering offers direct evidence for the excitonic instability of this phase transition in the ${\mathrm{Ta}}_{2}$ ${\mathrm{NiSe}}_{5}$ monolayer. The most prominent feature at low frequencies is a substantial enhancement of the Raman conductivity $\chi^{\prime \prime }/\omega$ near $T_{c}∼$ 190 K in the ac configuration, but not in the aa configuration (Figure 2a). This broad continuum of electronic excitations emerges with increasing temperature, reaches its maximum intensity at ∼ $T_{c}$, and then diminishes above $T_{c}$. Moreover, the phonon mode 2, corresponding to $B_{\text{2g}}$ modes in the high‐temperature orthorhombic phase, becomes highly asymmetric in the line shape above $T_{c}$, indicating strong Fano resonance with exciton continuum (Figure 2d). Additional monolayer ${\mathrm{Ta}}_{2}$ ${\mathrm{NiSe}}_{5}$ flakes, prepared from the same single crystal under identical procedures and conditions, exhibit qualitatively identical Raman spectra and similar temperature dependences (Figure S16). We fit the Raman conductivity 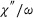 spectra, taking into account two contributions of the electronic (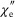) and phononic (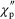) parts. The electronic part 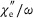 is described by the damped harmonic oscillator model as expressed by 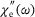 = $\frac{A_{e}\Gamma_{e}\omega }{\Gamma_{e}^{2}+\omega^{2}}$ while the phononic part 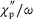 with the Fano lineshape is expressed as 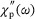 = $\frac{A_{\text{ph}}{\left(1+q\varepsilon \right)}^{2}}{1+\varepsilon^{2}}$, where $\varepsilon =\frac{\omega -\omega_{\text{ph}}}{\Gamma_{\text{ph}}}$, $A_{e}$ and $\Gamma_{e}$ represent the amplitude and width of the broad continuum of electronic excitation, $A_{\text{ph}}$, $\omega_{\text{ph}}$, $\Gamma_{\text{ph}}$, and q indicate the amplitude, energy, width, and the asymmetry of the Raman mode 2, respectively. For bulk ${\mathrm{Ta}}_{2}$ ${\mathrm{NiSe}}_{5}$, it has been known that this method yields the qualitatively same results as the model of coupled exciton and phonon excitations [22]. As shown in Figure 2d and Figure S14, the Raman conductivity 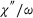 spectra below ∼ 100 ${\mathrm{cm}}^{-1}$ is well reproduced by this model.

We present the static susceptibility χ(T), obtained by integrating the electronic Raman conductivity 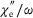 over the whole energy range except the sharp phonon contributions, in Figure 2f, together with 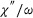 at ω = 7 ${\mathrm{cm}}^{-1}$, the lowest energy available in this experiment. Both the static susceptibility χ and the low energy 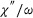 exhibit a clear maximum at $∼T_{c}$ estimated from the temperature‐dependent intensity of phonon mode 5 (Figure 2f). These features can be understood in terms of the Curie‐Weiss behavior of χ [45, 46, 47, 48] and its divergence at $T_{c}$ due to critical fluctuations of excitonic instability in the $B_{\text{2g}}$ channel, as intensively discussed in the bulk cases [22, 26, 49]. The best fit to the Curie–Weiss model, described by $\chi \left(T\right)\propto \frac{1}{T-\Theta }$, yields the Weiss temperature Θ ∼ 100 K, somewhat lower than the $T_{c}$ ∼ 190 K (Figure 2f). This difference between $T_{c}$ and Θ has been attributed to significant exciton‐phonon coupling, which raises $T_{c}$ associated with a structural transition above the otherwise putative electronic transition at Θ [22, 26, 49]. These observations provide compelling evidence that excitonic instability and its critical fluctuations persist even in a monolayer ${\mathrm{Ta}}_{2}$ ${\mathrm{NiSe}}_{5}$ and likely play an essential role in the observed phase transitions.

### 2.3 Thickness Dependence

**FIGURE 3:**
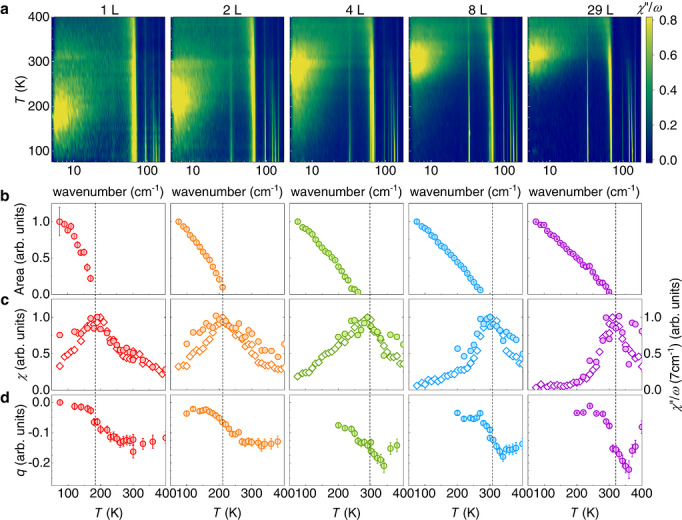
Systematic thickness dependence of phase transition. (a) False color map showing the normalized Raman conductivity in the ac configuration from 75 to 400 K for 1‐, 2‐, 4‐, 8‐, and 29‐layer ${\mathrm{Ta}}_{2}$ ${\mathrm{NiSe}}_{5}$. (b) Normalized integrated area of the phonon mode 5 as a function of temperature. (c) Normalized Raman conductivity at 7 ${\mathrm{cm}}^{-1}$ (diamond) and normalized static susceptibility (circle) as a function of temperature. (d) Asymmetry parameter q extracted from a Fano fit to phonon mode 2. The dashed vertical lines in b ‐ d indicate $T_{c}$ for each thickness.

**FIGURE 4:**
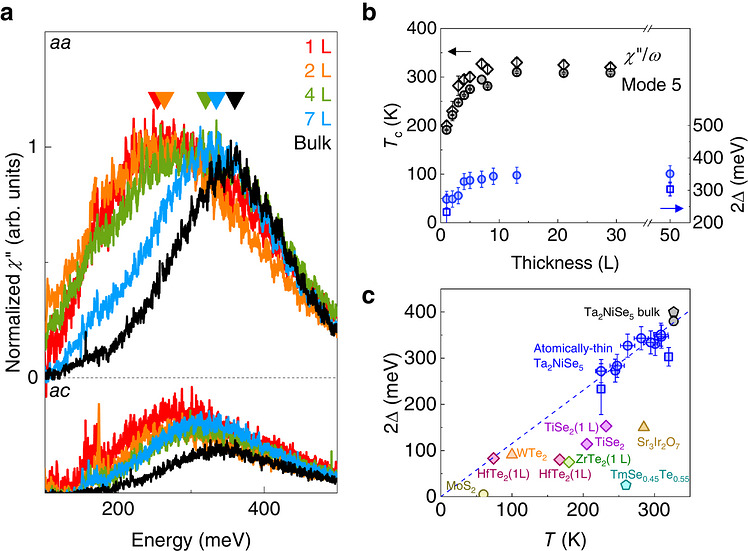
Thickness independent gap ratio. (a) Raman spectra in the high energy region with various thicknesses at 75 K in the aa and ac configuration. The spectra measured in the ac configuration are offset for clarity. Each color's triangles indicate the curve's maximum points. (b) Thickness dependent transition temperature obtained from Raman conductivity maximum (diamond) and integrated area of phonon mode 5 (circle). Hybridized gap size obtained from Raman spectroscopy (blue circle) and STM (square) show the same thickness dependence as $T_{c}$. (c) The hybridized gap size of excitonic insulator candidates as a function of the transition temperature. Different methods have been used to measure 2Δ, including Raman spectroscopy (circles), STM/STS (square), optical absorption (pentagon), and angle‐resolved photoemission spectroscopy (diamonds) [26, 31, 51, 52, 53, 54, 55, 56, 57, 58, 59]. Data for monolayer samples are denoted by “(1L)” next to the compound name, while bulk samples are unlabeled. All points for various thicknesses of ${\mathrm{Ta}}_{2}$ ${\mathrm{NiSe}}_{5}$ collapse onto a single line (blue dashed line), implying a nearly thickness independent gap ratio.

Having established that the excitonic instability persists down to the monolayer limit, we now discuss its thickness dependence. The characteristic Raman signatures of ${\mathrm{Ta}}_{2}$ ${\mathrm{NiSe}}_{5}$ are systematically modified with thickness. For all thicknesses, ranging from a monolayer to 29 layers (29 L), we observed that the phonon modes with either $A_{g}$ or $B_{\text{2g}}$ symmetry in the high‐temperature orthorhombic structure are suppressed with increasing temperature and eventually disappear at $T_{c}$ in the ac and aa configurations, respectively, confirming the same structural transition as observed in bulk and monolayer (see Figure 3a and Figure S13c). Similar to the monolayer case, we selected mode 5 in the ac configuration, which exhibits the most discernible intensity across all thicknesses, and monitored its temperature‐dependent peak intensity by fitting the Raman spectra to the Lorentzian line shape (see Figure 3b). The estimated $T_{c}$ ∼ 310 K of 29 L, at which the peak of phonon mode 5 disappears, aligns well with previous studies on bulk ${\mathrm{Ta}}_{2}$ ${\mathrm{NiSe}}_{5}$ [22, 26, 29, 40]. It remains nearly constant at approximately 310 K as the thickness decreases, but it rapidly decreases below 4 L, reaching ∼190 K for the monolayer (see Figures 3b and 4b and Figure S15b).

Consistently, the diverging behavior in the Raman conductivity 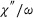 at low frequencies systematically shifts to lower temperatures as the thickness is reduced (see Figure 3a and Figure S15). Using the same model with electronic and phononic contributions as discussed above, we fitted the Raman conductivity 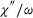 spectra of the samples with different thicknesses at each temperature (see Figure S14) to estimate the electronic susceptibility χ(T). The obtained χ(T), in good agreement with 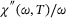 at $\omega =7{\text{cm}}^{-1}$, exhibits a clear peak at $T_{c}$ for all thicknesses, although it becomes broader with lowering thickness (see Figure 3c and Figure S15c). For all the nanoflakes with different thicknesses, the phase transition temperature extracted from the phononic signature (disappearance of mode 5) and from the electronic Raman response (peaks in χ(T) and 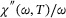 at $\omega =7{\text{cm}}^{-1}$) are in good agreement. Moreover, Fano resonance behavior of $B_{\text{2g}}$ phonon also shows a mild thickness dependence. We found that the asymmetry in the Raman spectra of phonon mode 2 due to Fano resonance with exciton continuum becomes pronounced above $T_{c}$ for all thicknesses. The lineshape of Raman peak for phonon mode 2 remains asymmetric with lowering thickness, in addition to the blue shift of the corresponding frequency. The best fit to the Fano resonance model consistently yields an asymmetry parameter q reduced with lowering thickness (Figure 3d and Figure S15d), indicating weak suppression of exciton‐phonon coupling. Clearly, the characteristic features of excitonic instability persist even approaching the monolayer limit, albeit systematically suppressed. These signatures are entirely absent in ${\mathrm{Ta}}_{2}$ ${\mathrm{NiS}}_{5}$ (Figure S17), where no excitonic instability is observed, and thus strongly support the presence of excitonic instability in ${\mathrm{Ta}}_{2}$ ${\mathrm{NiSe}}_{5}$.

**FIGURE 5:**
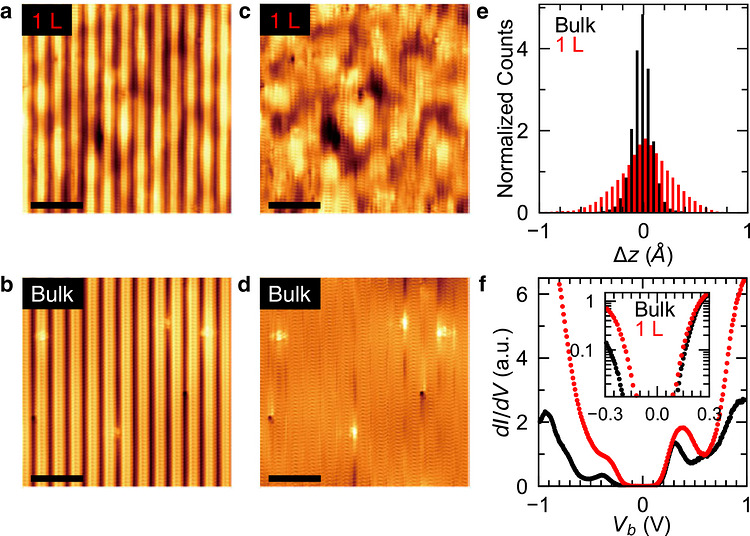
Effect of strain‐induced disorder in monolayer ${\mathrm{Ta}}_{2}{\mathrm{NiSe}}_{5}$. (a, b) Atom‐resolved scanning tunneling microscopy (STM) images of monolayer ${\mathrm{Ta}}_{2}{\mathrm{NiSe}}_{5}$ (a) and its bulk counterpart (b) obtained at 87 K. Scale bar, 5 nm. Imaging parameters: (a) 500 mV, 100 pA; (b) 700 mV, 100 pA. (c, d) Flattened STM topographic images of the monolayer and bulk surfaces, extracted from (a) and (b), highlighting surface irregularities arising from the underlying ${\mathrm{Al}}_{2}O_{3}$ substrate. (e) STM height histograms obtained from (c) and (d). Estimated roughness values are 22 pm for 1 L and 7.8 pm for bulk. (f) Differential tunneling conductance (dI/dV) spectra of monolayer and bulk ${\mathrm{Ta}}_{2}$ ${\mathrm{NiSe}}_{5}$. The inset shows the zoom‐in dI/dV, plotted on log scale. Gap sizes are 233 meV for 1 L and 303 meV for bulk.

Coherent hybridization gap opening driven by excitonic instability is also systematically changed, as confirmed by Raman spectroscopy at high frequencies. When the hybridization gap develops due to the coherent mixing of Ta 5d conduction and Ni 3d conduction bands, symmetry‐breaking excitations in the ac configuration are cancelled out by destructive interference effects [26]. In contrast, in the aa configuration, only fully symmetric excitations are detected and thus the interband transition produces significant excitation intensity due to a slightly different coupling between the two bands. As observed in the bulk, the Raman spectra of monolayer and few‐layer ${\mathrm{Ta}}_{2}$ ${\mathrm{NiSe}}_{5}$ crystals at 75 K show 3–5 times higher intensity in the aa configuration than in the ac configuration (Figure 4a), indicating the band hybridization below the excitonic transition. This hybridization gap Δ, estimated from the broad peak position of the Raman spectra, is 2Δ∼ 380 meV in the 29 L sample, consistent with that (∼303 meV) estimated from the scanning tunneling microscopy and spectroscopy (STM/STS) (Figure 5f) and the bulk values reported previously [26, 31, 32]. With lowering thickness, 2Δ remains nearly constant, but below ∼4 L it decreases systematically to ∼280 meV in the monolayer, which resembles the trend of $T_{c}$ with thickness (Figure 4b). The decreased gap size in the monolayer is also observed by STM (∼233 meV, Figure 5f). Consequently, 2Δ and $T_{c}$ follow the linear dependence with a nearly constant gap ratio 2Δ/$k_{B}$ $T_{c}$ ∼ 13 (Figure 4c), where $k_{B}$ is the Boltzmann constant. This gap ratio is well above the weak‐coupling BCS limit of ∼3.5 [50], similar to that obtained for the bulk and markedly larger than those of other excitonic‐insulator candidates, including monolayer ${\mathrm{WTe}}_{2}$ [51], ${\mathrm{HfTe}}_{2}$ [52], ${\mathrm{ZrTe}}_{2}$ [53], and ${\mathrm{TiSe}}_{2}$ [54], as well as bulk ${\mathrm{Sr}}_{3}$ ${\mathrm{Ir}}_{2}$ $O_{7}$ [55, 56], ${\mathrm{TmSe}}_{0.45}$ ${\mathrm{Te}}_{0.55}$ [57], ${\mathrm{TiSe}}_{2}$ [58], and ${\mathrm{MoS}}_{2}$ [59] (Figure 4c). These findings clearly suggest that strong coupling nature of excitonic instability is preserved in atomically‐thin ${\mathrm{Ta}}_{2}$ ${\mathrm{NiSe}}_{5}$.

### 2.4 Strain Disorder in Monolayer ${\mathrm{Ta}}_{2}$ ${\mathrm{NiSe}}_{5}$

To understand the reduction of $T_{c}$ in the monolayer relative to the bulk, we carried out STM and STS measurements on both monolayer and bulk ${\mathrm{Ta}}_{2}$ ${\mathrm{NiSe}}_{5}$. One possible origin of the reduced $T_{c}$ is charged disorder, such as defects introduced during the exfoliation procedure or charge impurities in the underlying substrate, which can strongly suppress the excitonic instability and thereby lower $T_{c}$ [60]. By analogy with BCS superconductivity, charged disorder in excitonic insulators may play a role similar to that of paramagnetic impurities in superconductors, causing asymmetric scattering of the paired constituents and thereby inducing a pair‐breaking effect [61]. However, our STM/STS measurements on monolayer ${\mathrm{Ta}}_{2}$ ${\mathrm{NiSe}}_{5}$, taken at ∼87 K, show a defect density nearly identical to that of the bulk (Figure 5a,b). Moreover, we observed improved charge balance in the monolayer, with the chemical potential located at the center of the gap, as confirmed by the STS spectra (Figure 5f). These observations rule out charged disorder as the primary cause for the observed reduction in $T_{c}$ (Figure 4b).

In contrast, strain disorder, characterized by a random quenched distribution of local strain within the layer, is not expected to disrupt the charge balance between electrons and holes. Analogous to nonmagnetic impurities in BCS superconductivity, such disorder is expected to have only a minimal effect on $T_{c}$ in the case of a purely excitonic instability. However, in exciton‐lattice‐coupled systems, strain can alter phonon characteristics, such as mode frequencies and dispersions, and thus significantly affect the critical fluctuations of the exciton order parameter, as evidenced by the suppression of $T_{c}$ under tensile or compressive strain in bulk ${\mathrm{Ta}}_{2}$ ${\mathrm{NiSe}}_{5}$ [62]. Therefore, strain disorder is expected to generate spatial inhomogeneity in the exciton‐phonon coupling strength, thereby broadening and suppressing the excitonic phase transition. Indeed, we found that surface roughness, estimated from the height histogram of flattened STM topographic images, becomes approximately three times larger in the monolayer than in the bulk (Figure 5c,d). This increase arises because the monolayer conforms to the surface corrugation of the underlying ${\mathrm{Al}}_{2}$ $O_{3}$ substrate (Figure 5e). The presence of strain disorder is further supported by the systematic increase of the full‐width‐at‐half‐maximum (FWHM) of all the Raman peaks with reducing thickness (Figure S11).

Consistently, the hallmarks of exciton‐phonon coupling exhibit pronounced thickness dependence, i.e. the peak in the temperature‐dependent electronic susceptibility χ(T) broadens (Figure 3c), and the Fano asymmetry parameter q of the phonon mode weakens with decreasing thickness (Figure 3d). By contrast, such strain disorder is unlikely to induce substantial changes in phonon‐phonon interactions in ${\mathrm{Ta}}_{2}$ ${\mathrm{NiSe}}_{5}$. For all samples investigated, down to the monolayer limit, we observe neither a signature of phonon softening nor a significant change in the phonon‐phonon interaction strength, as extracted from the temperature dependence of the phonon mode frequencies below $T_{c}$ using the anharmonic phonon decay model (Figure S12). Taken together, these results suggest that strain inhomogeneity in monolayer ${\mathrm{Ta}}_{2}$ ${\mathrm{NiSe}}_{5}$ modulates $T_{c}$ primarily via exciton‐phonon coupling rather than through changes in phonon‐phonon interactions. This interpretation is also consistent with density‐functional‐theory calculations that include electron correlation, which show that the calculated gap size correlates with tensile and compressive strain along the Ta‐Ni‐Ta chains (Figure S7).

## 3 Discussion

Our key findings are the very presence of an excitonic instability at the monolayer limit and the large gap ratio $2\Delta /k_{B}T_{c}∼13$ regardless of thickness reduction. The strong coupling nature in ${\mathrm{Ta}}_{2}$ ${\mathrm{NiSe}}_{5}$, despite the semimetal‐type non‐interacting band structures [63], has been attributed to a significant electron‐phonon coupling, in addition to the strong interband Coulomb interaction for exciton formation [21, 24, 27, 39, 64, 65]. The clear Fano signature with $B_{\text{2g}}$ phonon modes [22, 26, 29, 40] observed via Raman spectroscopy in Figure 2, Fano resonance in the infrared optical spectra [31, 39], and softening of $B_{\text{2g}}$ acoustic phonon modes probed by inelastic X‐ray scattering [21] confirm that $B_{\text{2g}}$ acoustic and optical phonon modes, associated with opposite tilting of adjacent Ta chains in the Ta‐Ni‐Ta ladders (Figure 1c), are strongly coupled to the exciton continuum above $T_{c}$ in ${\mathrm{Ta}}_{2}$ ${\mathrm{NiSe}}_{5}$. In the presence of such exciton‐phonon coupling, the excitonic phase transition is no longer described by continuous U(1) symmetry breaking but rather by $Z_{2}$ symmetry breaking [66, 67]. The resulting Goldstone modes acquire a substantial low‐energy gap, which is crucial for stabilizing a long‐range order at the 2D limit against thermal fluctuations at high temperatures.

The strong coupling nature of the excitonic instability further provides an essential premise for understanding the observed decrease of $T_{c}$ with thickness reduction. In the strong coupling regime, the spatial extension of the exciton shrinks significantly and becomes comparable with the interlayer spacing [68]. In fact, the estimated spatial extent of the exciton is highly anisotropic and strongly confined within the Ta‐Ni‐Ta ladders, with an exciton Bohr radius of ∼ 3 Å normal to the chain direction [25, 69]. This contrasts with other vdW semiconductors, e.g. ${\mathrm{MoS}}_{2}$ [4] in which excitons typically have a Bohr radius of ∼10 nm, an order of magnitude larger than in ${\mathrm{Ta}}_{2}$ ${\mathrm{NiSe}}_{5}$, and they show a drastic enhancement of their binding energy by a factor of ∼5‐15 from bulk to monolayer [4, 68, 70]. In ${\mathrm{Ta}}_{2}$ ${\mathrm{NiSe}}_{5}$, a slight suppression of dielectric screening with lowering thickness is indirectly inferred from the blueshifts found in most phonon modes (Figure S10). However, its impact on the interband Coulomb interaction and the excitonic instability appears to be insufficient to overcome the strain disorder effect, resulting in a mild reduction of $T_{c}$.

One remaining question is the detailed role of strain disorder in exciton‐phonon coupling in ${\mathrm{Ta}}_{2}$ ${\mathrm{NiSe}}_{5}$. Upon reducing thickness, the coupling strength of $B_{\text{2g}}$ Raman phonon with exciton continuum decreases (Figure 3d), while the large gap ratio of $2\Delta /k_{B}T_{c}$ remains nearly intact (Figure 4c). This seeming contradiction can be attributed to the distinct contributions of low‐energy acoustic and high‐energy optical phonons to the gap ratio and $T_{c}$ in ${\mathrm{Ta}}_{2}$ ${\mathrm{NiSe}}_{5}$. It has been well established for electron‐phonon coupled superconductors that for a given area of electron‐phonon coupling spectrum $\alpha^{2}F\left(\omega \right)$, low frequency spectrum predominantly determines the gap ratio $2\Delta /k_{B}T_{c}$, whereas $T_{c}$ is significantly affected by the high frequency spectrum, with a maximum at ω ∼ 7$k_{B}T_{c}$ [71]. Similarly, in monolayer ${\mathrm{Ta}}_{2}$ ${\mathrm{NiSe}}_{5}$, $B_{\text{2g}}$ acoustic phonon modes is less influenced by strain disorder and their coupling to the exciton continuum is mostly responsible for the large gap ratio. In contrast, the strong coupling with $B_{\text{2g}}$ optical phonon modes is critical for enhancing $T_{c}$, but it is more susceptible to strain disorder. While further investigations is desirable particularly about mode‐specific exciton‐phonon coupling strength, these results suggest that strain manipulation is the key to achieving a high temperature electronic instability due to exciton‐phonon coupling in monolayer ${\mathrm{Ta}}_{2}$ ${\mathrm{NiSe}}_{5}$.

Our findings reveal the collaborative interplay between strong interband Coulomb interaction and electron‐phonon coupling in the monolayer ${\mathrm{Ta}}_{2}$ ${\mathrm{NiSe}}_{5}$. When compared to other excitonic insulator candidates, the monolayer ${\mathrm{Ta}}_{2}$ ${\mathrm{NiSe}}_{5}$ is well within the strong coupling regime (Figure 4c), making it an ideal 2D system for investigating and harnessing the correlated exciton‐phonon states under external stimuli, such as an external electric field in the field‐effect transistor geometry, uniaxial strain using a flexible substrate, or proximity coupling by heterostructuring with other 2D materials. We envision that these versatile controls, readily accessible for 2D materials, allows us to address various proposed properties of excitonic insulators [17, 18, 19]. Furthermore, given that the limiting factor for $T_{c}$ is strain disorder, reducing surface roughness through improved fabrication techniques, such as hBN encapsulation [72], holds the potential to further enhance $T_{c}$ in the monolayer of ${\mathrm{Ta}}_{2}$ ${\mathrm{NiSe}}_{5}$. Recently, $T_{c}$ enhancement up to ∼380 K was reported for the monolayer ${\mathrm{Ta}}_{2}$ ${\mathrm{NiSe}}_{5}$ encapsulated with atomically‐flat hBN layers, where strain disorder is expected to be smaller than our samples [73]. These findings suggest that strong exciton‐phonon coupling has dual effects, which enhance the excitonic instability at elevated temperatures, while also making the transition highly sensitive to strain‐induced disorder. These characteristics offer a route for tuning the excitonic instability through strain engineering while highlighting the need to control local strain fluctuations when designing devices based on monolayer ${\mathrm{Ta}}_{2}$ ${\mathrm{NiSe}}_{5}$.

## 4 Experimental Section

### 4.1 Single Crystal Growth and Exfoliation

High‐quality single crystals of ${\mathrm{Ta}}_{2}$ ${\mathrm{NiSe}}_{5}$ were synthesized through a chemical vapor transport method. A mixture of Ta, Ni, and Se powders, with a stoichiometry ratio of Ta: Ni: Se = 2: 1: 5+α, were placed in an evacuated quartz tube and sintered at a temperature of 900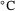 for 7 days. Then the synthesized ${\mathrm{Ta}}_{2}$ ${\mathrm{NiSe}}_{5}$ poly crystals were sealed in an evacuated quartz tube again with iodine as a transport agent. The tube was placed in a furnace with a temperature gradient between 960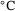 and 850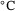 for 2 weeks. Single crystals with typical size of 0.1 mm × 1 mm × 20 mm are obtained. The crystallinity and stoichiometry of the crystals were confirmed by X‐ray diffraction and energy‐dispersive spectroscopy.

In order to isolate monolayer and few‐layer ${\mathrm{Ta}}_{2}$ ${\mathrm{NiSe}}_{5}$ from the bulk crystal, we followed the ${\mathrm{Al}}_{2}$ $O_{3}$‐assisted exfoliation method as described in ref. [41] and Figure S1. The isolated monolayer and few‐layer ${\mathrm{Ta}}_{2}$ ${\mathrm{NiSe}}_{5}$ were transferred to a sapphire substrate as shown in the optical transmission images (Figure 1b and Figure S2a). The number of layers was determined using the optical transmittance $G_{s}$ and $G_{\text{sub}}$ obtained from the green channel transmission intensity through the sample and substrate. $G_{s}$/$G_{\text{sub}}$ follows the Beer–Lambert law as a function of the number of layers, which allowed the thickness of the sample to be determined (Figure S2b). To prevent degradation in air, the entire exfoliation and evaporation process was performed in an argon atmosphere with a humidity of less than 0.1 ppm or under vacuum. The transfer of the monolayer and few‐layer samples into a vacuum chamber for Raman spectroscopy measurements was also carried out in the Ar‐filled glove box ($H_{2}O$ < 0.1 ppm) to avoid any exposure to the air.

### 4.2 Polarization‐Resolved Raman Spectroscopy Measurements

The Raman spectra were measured using home‐built confocal microscopy set‐up (Figure S3). A He–Ne laser (632.8 nm) was used as the excitation source and propagates along the b crystallographic axis with laser beam size of ∼1 μm and laser power of ∼0.2 mW. The aa and ac configurations were defined by two linear polarizers and the polarization of the incident light relative to the crystal orientation was controlled by an achromatic half‐wave plate. Low‐energy signals (above 7 ${\mathrm{cm}}^{-1}$) could be investigated by using a set of grating‐based notch filters (Optigrate, ${\mathrm{BragGrate}}^{\text{TM}}$ notch filters) to remove elastic light. The Raman signal is collected in the backscattering geometry and analyzed by a 750‐mm monochromator and a liquid nitrogen‐cooled CCD (Princeton Instruments). The temperature of samples were controlled by using an optical cryostat (Oxford Instruments, Hires2) and the temperature at the laser spot was estimated by the Stokes and anti‐Stokes relation of phonons (Figure S4). At both high and low temperatures, the laser heating was estimated to be about 0.5 ∼ 5 K, which is similar to the error in the $T_{c}$ determination, so we neglected the laser heating.

While Raman spectroscopy is widely used to detect phonon modes, it can also probe electronic excitations via inelastic light scattering, known as electronic Raman scattering. Because electronic Raman scattering is sensitive to changes in the electronic excitation spectrum (e.g., gap opening or the emergence of collective modes), it can serve as an indicator of electronic phase transitions. In particular, its polarization dependence provides direct insight into the symmetry of excitation. The electronic Raman response forms a broad continuum, contrast to the sharp peak of phonon modes, therefore background correction is required to obtain an accurate electronic Raman signal from the sample. In our experiments, the underlying ${\mathrm{Al}}_{2}$ $O_{3}$ layers contribute a background Raman signal, characterized by a gradual increase in 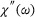 as the frequency decreases. To address this, we measured the background Raman signal from a nearby ${\mathrm{Al}}_{2}$ $O_{3}$‐only region and subtracted it from the spectra of the ${\mathrm{Ta}}_{2}$ ${\mathrm{NiSe}}_{5}$ nanoflakes as shown in Figure S5.

## Author Contributions

J.S.K. and S.Y.K. conceived the projects. S.Y.K. and J.H.Y. prepared the ultrathin layers. S.Y.K., K.K., J.H.Y., J.K., and B.J.K. conducted Raman spectroscopy and analysed the data. D.K. and T.‐H.K. performed scanning tunnelling microscopy/spectroscopy and data analysis. J.E.L., J.S., and J.H.K. conducted optical spectroscopy measurements and spectral analysis. C.‐J.K. performed the electronic‐structure calculations. C.I.K. synthesized the bulk crystals. J.K., H.W.Y., J.H.K., and B.J.K. contribute to the data analysis. S.Y.K., K.K., T.‐H.K., J.K., B.J.K., J.S.K. co‐wrote the manuscript. All authors discussed the results and commented on the paper.

## Conflicts of Interest

The authors declare no conflicts of interest.

## Supporting information


**Supporting File 1**: advs75569‐sup‐0001‐SuppMat.pdf.

## Data Availability

The data that support the findings of this study are available from the corresponding author upon reasonable request.
