# Supplementary material for: Observation of Excitonic Instability in a Monolayer Ta2NiSe5 With Strain Disorder
Source: Adv Sci (Weinh). 2026 May 11;13(43):e75569. doi: 10.1002/advs.75569 (PMC13336087; doi:10.1002/advs.75569)
Supplement: Supplementary file 1 — Supporting File: advs75569‐sup‐0001‐SuppMat.pdf. [file ADVS-13-e75569-s001.pdf]

# Observation of excitonic instability in a monolayer $Ta_2NiSe_5$ with strain disorder

So Young Kim,[1,2,*] Kwangrae Kim,[1,*] Dowook Kim,[1] Ji Eun Lee,[3] Jieun Seok,[3] Chang Il Kwon,[1,4] Jo Hyun Yun,[1,4] Chang-Jong Kang,[5] Jae Hoon Kim,[3] H. W. Yeom,[1,4] Tae-Hwan Kim,[1,†] Jonghwan Kim,[1,2,6,‡] B. J. Kim,[1,§] and Jun Sung Kim[1,4,¶]

[1]*Department of Physics, Pohang University of Science and Technology (POSTECH), Pohang 37673, Korea*

[2]*Department of Materials Science and Engineering, Pohang University of Science and Technology, Pohang 37673, Korea*

[3]*Department of Physics, Yonsei University, Seoul 03722, Korea*

[4]*Center for Artificial Low Dimensional Electronic Systems, Institute for Basic Science (IBS), Pohang 37673, Korea*

[5]*Department of Physics, Chungnam National University, Daejeon 34134, Korea*

[6]*Center for Van der Waals Quantum Solids, Institute for Basic Science (IBS), Pohang, 37673, Korea*

[*] equal contribution

[†] taehwan@postech.ac.kr

[‡] jonghwankim@postech.ac.kr

[§] bkim6@postech.ac.kr

[¶] js.kim@postech.ac.kr

**Supplementary Note 1: Experimental Set-up**

We used the $Al_2O_3$-assisted exfoliation method as described in ref. [1] to isolate monolayer and few-layer $Ta_2NiSe_5$ from the bulk crystal. In our previous work [2], we used a conventional exfoliation method using Scotch tape and obtained the thinnest sample with three-layer thick and a few-micrometer width. In order to obtained a monolayer with a large area, suitable for reliable Raman spectroscopy measurements, we had to employ the $Al_2O_3$-assisted exfoliation method. The procedure largely similar to that presented in Ref. [1] is as follows (Supplementary Figure 1).

(1) Evaporate $\sim$ 50 nm of $Al_2O_3$ onto the single crystal placed on tape.
(2) Attach a piece of thermal-release tape to the coated surface and then peel it off, then the exfoliated crystal layer(s) is obtained together with the deposited $Al_2O_3$.
(3) Place the thermal release tape and $Al_2O_3$/crystal layer(s) stack onto a Gel-Pak and apply heating to release the stack onto the Gel-Pak surface.
(4) Dry-transfer this to the target (sapphire) substrate with the sample surface facing upward.

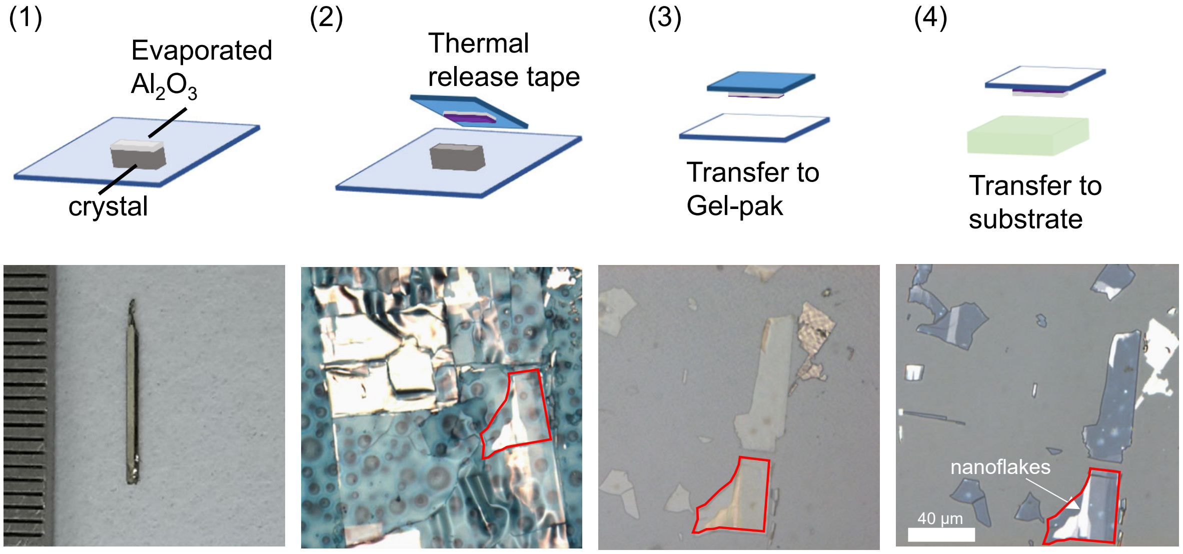


Supplementary Fig. 1: **Procedure of $Al_2O_3$-assisted exfoliation method.** Top row : schematics of each stpe of the procedure. Bottom row : images of the crystal attached to the tape or substrate at the corresponding steps.

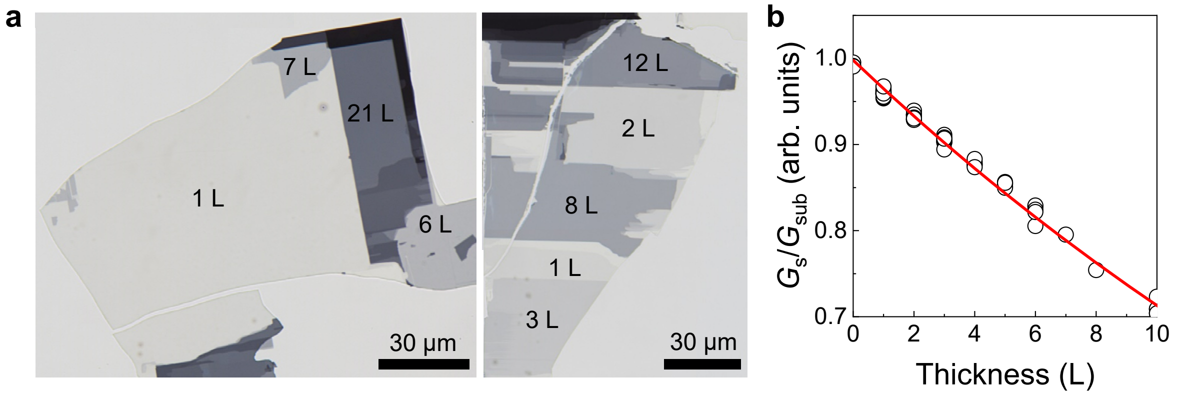


Supplementary Fig. 2: **Exfoliation and thickness determination.** **a**, Optical images of a typical exfoliated sample with different thicknesses, exfoliated by the $Al_2O_3$-assisted exfoliation method. The isolated monolayer and few-layer $Ta_2NiSe_5$ were transferred to a sapphire substrate. **b**, Relative optical transmittance of $Ta_2NiSe_5$ layers as a function of the thickness, following the Beer-Lambert law (red curve).

A typical exfoliated sample on a sapphire substrate obtained by this method is shown in Supplementary Fig. 2a. The optical transmittances of the sample and the bare substrate, $G_s$ and $G_{sub}$, were extracted from the green channel transmission intensity. The ratio $G_s/G_{sub}$ follows the Beer-Lambert law as a function of the number of layers, accordingly, we determined the sample thickness by fitting $G_s/G_{sub}$ to this model (Supplementary Fig. 2b).

The Raman spectra were measured with a home-built confocal Raman microscope (Supplementary Fig. 3). A He-Ne laser (632.8 nm) with beam size of $sim$ 1 $mu$m and laser power of $\sim$ 0.2 mW was incident along the crystallographic $b$-axis and the polarization-resolved spectra were collected in a backscattering geometry. The incident light was linearly polarized by a polarizer, and its orientation with respect to the crystal axes was controlled using an achromatic half-wave plate. With the incident polarization aligned along the crystal $a$-axis, we denote spectra recorded with the analyzer set parallel to the incident polarization as $aa$ and those recorded with the analyzer set perpendicular (along the $c$-axis) as $ac$. Low-frequency Raman shifts down to 7 cm$^{-1}$ could be investigated by using a set of grating-based notch filters (Optigrate, BragGrate$^{\text{TM}}$ notch filters) to suppress elastic light.

The temperature at the laser spot on the sample was estimated by the Stokes and anti-Stokes relation of phonons as described in ref. [3, 4]. For each system temperature $T_{sys}$, we computed a calculated temperature $T_{calc}$ by determining the temperature at which $\omega^3$-

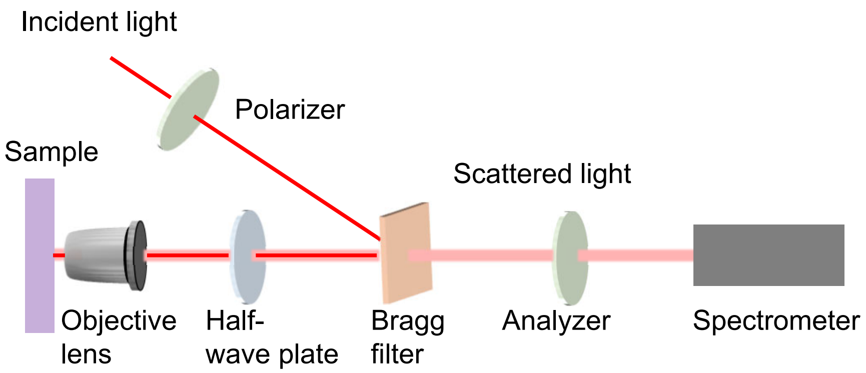


Supplementary Fig. 3: **Experimental configuration of polarized Raman measurements.**

corrected anti-Stokes intensity of a given phonon mode equals the corresponding Stokes intensity (Supplementary Figs. 4a and 4c). When using a CCD as the detector, the detected Raman intensity is proportional to $\omega^3$, where $\omega$ is the frequency of the scattered photon. Therefore, when comparing the Stokes and anti-Stokes spectra, the spectra must be corrected by dividing by $\omega^3$ [4]. The estimated sample temperature $T_{est}$ was then defined as the average of the $T_{calc}$ values at $T_{sys}$. The close agreement between the corrected Stokes and anti-Stokes spectra (Supplementary Figs. 4b and 4d) confirms that the $T_{est}$ reliably captures that the laser-induced temperature rise is small on the order of $0.5 - 5$ K.

While Raman spectroscopy is widely used to detect phonon modes, it can also probe electronic excitations via inelastic light scattering, known as electronic Raman scattering. Because electronic Raman scattering is sensitive to changes in the electronic excitation spectrum (e.g., gap opening or the emergence of collective modes), it can serve as an indicator of electronic phase transitions. In particular, its polarization dependence provides direct insight into the symmetry of excitation. The electronic Raman response forms a broad continuum, contrast to the sharp peak of phonon modes, therefore background correction is required to obtain an accurate electronic Raman signal from the sample. In our experiments, the underlying Al2O3 layers contribute a background Raman signal, characterized by a gradual increase in $\chi''(\omega)$ as the frequency decreases. To address this, we measured the background Raman signal from a nearby $Al_2O_3$-only region and subtracted it from the spectra of the $Ta_2NiSe_5$ nanoflakes as shown in Supplementary Fig. 5. After applying this correction, we observed good agreement between the $\chi''(\omega)$ spectra of a relatively thick sample (13L) and

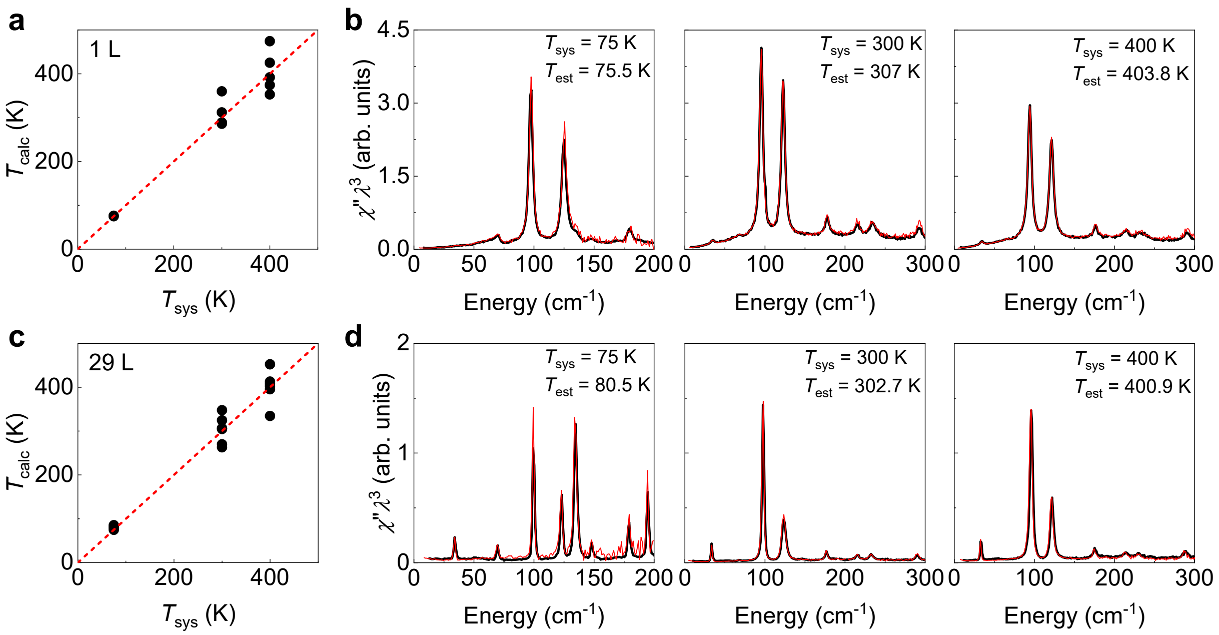


Supplementary Fig. 4: **Estimation of temperature at laser spot.** The temperature at laser spot is estimated from the Stokes and anti-Stokes relation of phonons. **a** and **c** show $T_{\text{calc}}$ as a function of $T_{\text{sys}}$ in 1 L and 29 L, respectively. The red dashed line indicates $T_{\text{sys}} = T_{\text{calc}}$ for guidance. **b** and **d** are the corrected Stokes scattering (black) spectra and anti-Stokes scattering spectra (red) of 1 L and 29 L samples. $\lambda$ represents the wavelength of the scattered photon.

bulk $Ta_2NiSe_5$, confirming the validity of our data analysis. The same procedure was applied to thinner flakes down to the monolayer (1L). As representative examples, we presented the measured Raman spectra for $Ta_2NiSe_5$ nanoflakes with thicknesses of 1L, 9L, and 13L in Supplementary Fig. 5. The estimated electronic gap slightly decreases for the 9L sample but shows a significant reduction for the 1L sample. We note that the reduced gap derived from the Raman spectra agrees well with that observed in scanning tunneling spectroscopy, as shown in Fig. 5f of the main text.

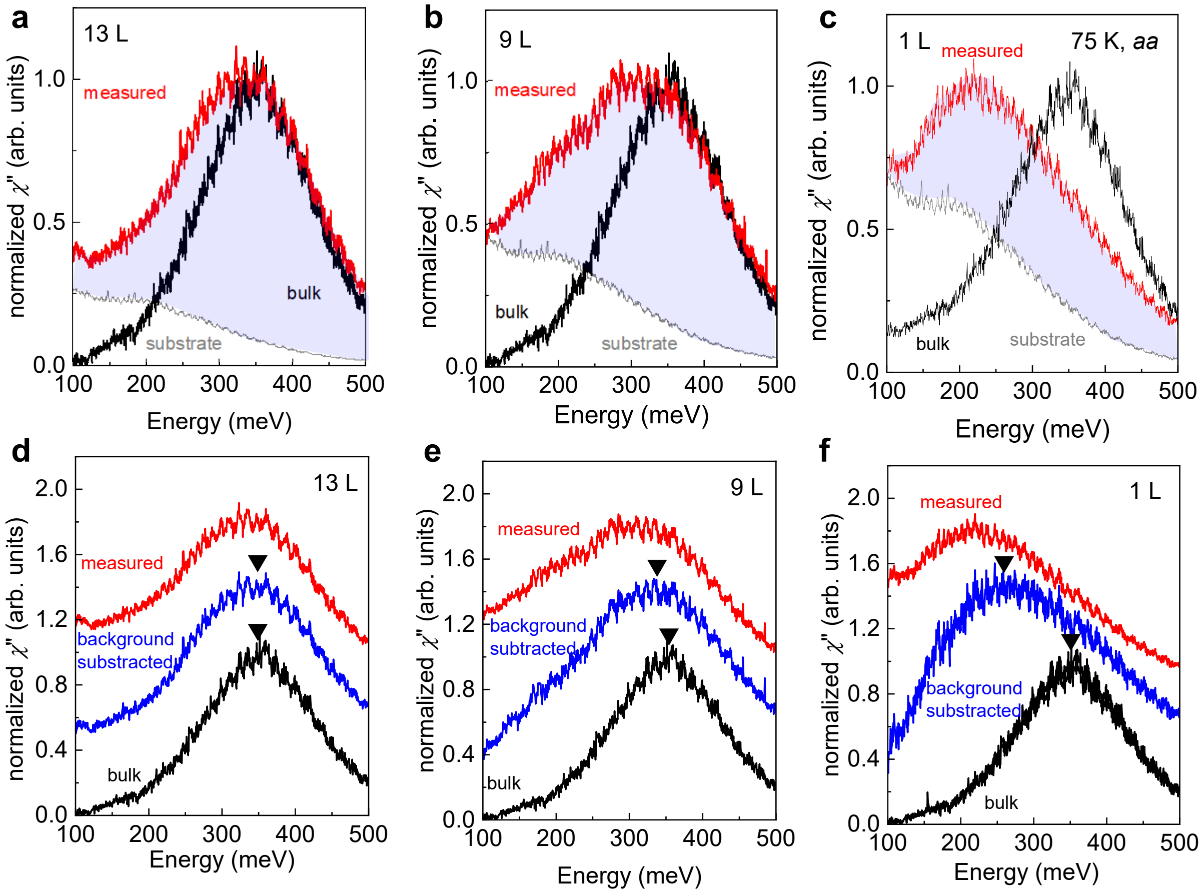


Supplementary Fig. 5: **Raman spectra of $Ta_2NiSe_5$ nanoflakes. a-c**, Raman susceptibility spectra ($\chi''$) in the *aa* configuration measured at 75 K for three representative nanoflakes with thicknesses of 13L (**a**), 9L (**b**), and 1L (**c**). The Raman spectra obtained from $Ta_2NiSe_5$ nanoflakes (red) are compared with those taken from a nearby $Al_2O_3$-only region (gray) and with bulk $Ta_2NiSe_5$ (black). The blue-shaded area highlights the contribution from the nanoflakes alone. **d-f**, Corresponding Raman spectra before (red) and after (blue) subtracting the background signal from the $Al_2O_3$ layers. For comparison, the bulk spectra are also displayed. The estimated electronic gap is marked by downward triangles in **d-f**.

**Supplementary Note 2: Electronic band structure**

We performed density functional theory (DFT) calculations to investigate the electronic structure of monolayer $Ta_2NiSe_5$. Experimentally characterizing the electronic structure of a monolayer $Ta_2NiSe_5$ is highly challenging due to the small size of the monolayer sample relative to the typical beam size used in angle-resolved photoemission spectroscopy (ARPES), which will require future studies. Previous DFT calculations for bulk $Ta_2NiSe_5$ have shown that accurately reproducing the experimentally observed electronic gap, both above and below $T_c$, is also difficult. According to ARPES and scanning tunneling spectroscopy experiments [5–7], a clear electronic gap of $\sim 0.3$ eV forms below $T_c$ in the low-temperature monoclinic phase. At high temperatures above $T_c$, the gap reduces but remains finite at $\sim$ 0.1 eV. However, many DFT calculations, using common exchange-correlation functionals such as GGA, Perdew-Burke-Ernzerhof (PBE), and modified Becke-Johnson (mBJ), tend to predict band structures with gapless semi-metallic type or with a much smaller gap than experimentally observed, unless on-site Coulomb interactions (typically $U \sim 2$ eV for Ta $d$-orbitals and U $\sim 5$ eV for Ni $d$-orbitals) are included.

Recently we found that using the recently-developed $r^2$SCAN functional with non-local van der Waals (rVV10) corrections allowed us to reproduce an insulating band structure with the characteristic $M$-shaped dispersions below $T_c$ for the monoclinic phase, even without including a finite on-site $U$. However, the calculated gap size ($\sim 0.1$ eV) remains significantly smaller than the experimental values, and the gap completely disappears above $T_c$ in the orthorhombic phase. These results indicate that while structural changes below $T_c$ contribute to the gap opening, strong electron correlations with excitonic instability, are crucial for accurately reproducing the large size of electronic gap, experimentally measured. We note that this observation is consistent with the recent study using pump-probe Raman and photoluminescent spectroscopy on $Ta_2NiSe_5$ revealing that electronic gap is nearly collapsed in a metastable state, where monoclinic lattice distortion is preserved but electron correlation is reduced by screening of photoexcited carriers [8].

Having clarified the importance of the electron correlation for gap opening, we calculated band structures of $Ta_2NiSe_5$ layers from monolayer (1L) to four layers (4L) and compared them with the bulk case. In all cases, structures are optimized with $r_2$SCAN and the non-local vdW correction scheme. As shown in Supplementary Figure 6, the $M$-shaped electronic

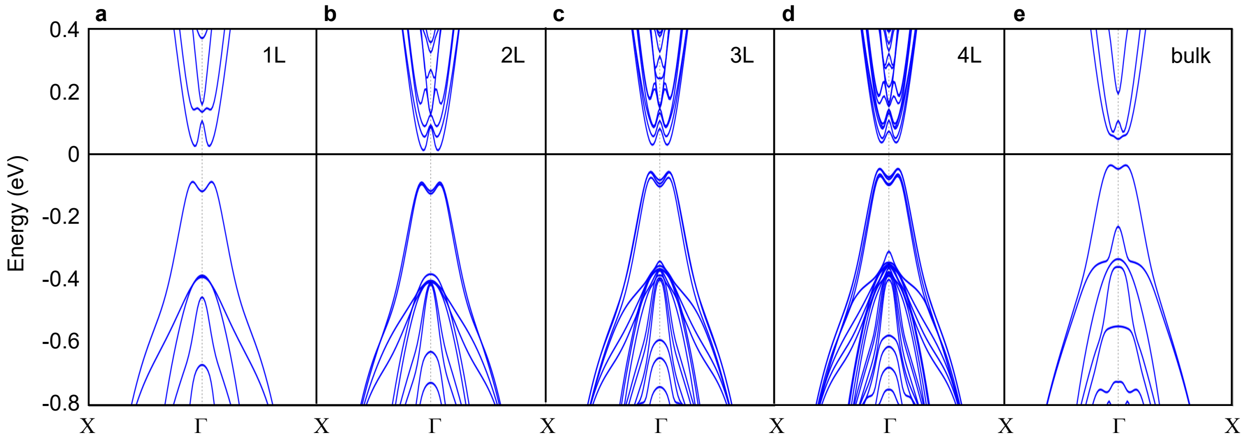


Supplementary Fig. 6: **Electronic structures of monoclinic $Ta_2NiSe_5$ with variation of thickness.** The typical M-shaped structures in both valence and conduction bands are commonly observed for all thickness from monolayer (1L) to bulk with a similar electronic gap of $\sim$ 0.1 eV.

structures and an energy gap of $\sim$ 0.1 eV remain nearly the same for all thicknesses. This aligns well with optical spectra indicating that the electronic structure remains essentially unchanged with varying thickness (Supplementary Fig. 8). These calculations indicate that the non-interacting band structure of a band-overlap semimetal in monolayer. In conventional understanding on excitonic insulators, such a semimetal band structure favors weak coupling, described analogously by the Bardeen–Cooper–Schrieffer (BCS) theory. However, the large gap ratio $\Delta/k_B T_c \sim 13$, observed in Raman spectroscopy and STS, as well as non-metallic resistivity with a finite gap above $T_c$ as shown in Supplementary Fig. 18 provide compelling evidence that monolayer $Ta_2NiSe_5$ lies in the strong-coupling regime. This means that the interband hybridization due to excitonic instability is enhanced by significant exciton-phonon coupling in $Ta_2NiSe_5$, and thus Bose-Einstein condensation of preformed excitons occur even on the semimetal side, as demonstrated in a recent theoretical study [9]. These additional electronic structure calculations consistently support our main conclusion of essential role of excitonic instability with significant exciton-phonon coupling.

In order to address the strain effect on phase transition in monolayer $Ta_2NiSe_5$, we calculated the electronic structures with compressive and tensile strain along the $a$ or $c$ axes corresponding to parallel or perpendicular direction to the Ta-Ni-Ta chains. As discussed above, we employed the recently-developed r$^2$SCAN functional with non-local van der Waals

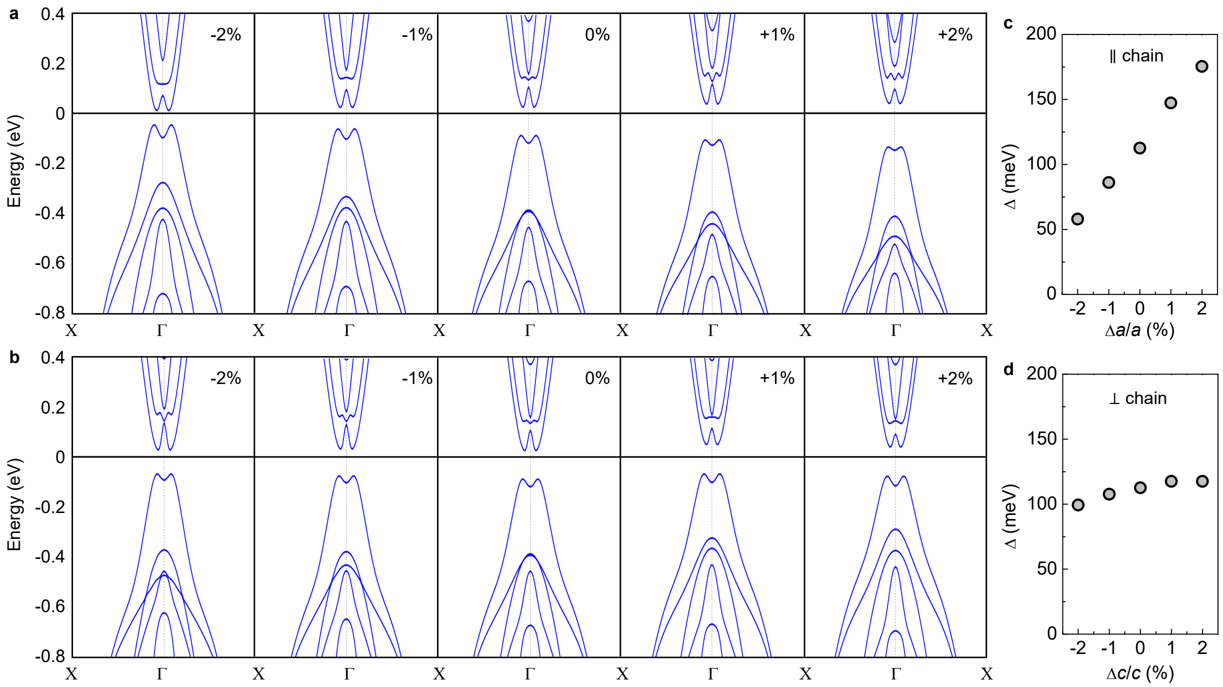


Supplementary Fig. 7: **Strain dependent electronic structures of monolayer $Ta_2NiSe_5$. a**, **b**, Typical M-shaped band structures of monolayer $Ta_2NiSe_5$ in monoclinic phase, with variation of uniaxial strain for parallel (**a**) and perpendicular (**b**) to the Ta-Ni-Ta chains. The relative changes of the lattice parameters $\Delta a/a$ (**a**) or $\Delta c/c$ (**b**) are indicated. **c, d**, Strain dependent energy gap $\Delta$ for parallel (**c**) and perpendicular (**d**) to the Ta-Ni-Ta chains.

(rVV10) corrections, which reproduce an insulating band structure with the characteristic $M$-shaped dispersions below $T_c$ for the monoclinic phase. We considered uniaxial strain with modulation of $a$ or $c$ parameters and the other lattice parameter remains the same. While strong electron correlations with excitonic instability is not considered and thus the gap size is underestimated as compared to the experimental value, these calculations reveal that the strain does affect significantly to the interband hybridization due to monoclinic distortion. As shown in Supplementary Fig. 7, the energy gap is dramatically enhanced and suppressed with a few % of tensile and compressive strains along the Ta-Ni-Ta chains, respectively, while it remains almost same with strains perpendicular to the chains. Combining with our STS results discussed in the main text, these calculations suggest that strain effect should be taken into account to understand thickness dependence of the phase transition in $Ta_2NiSe_5$.

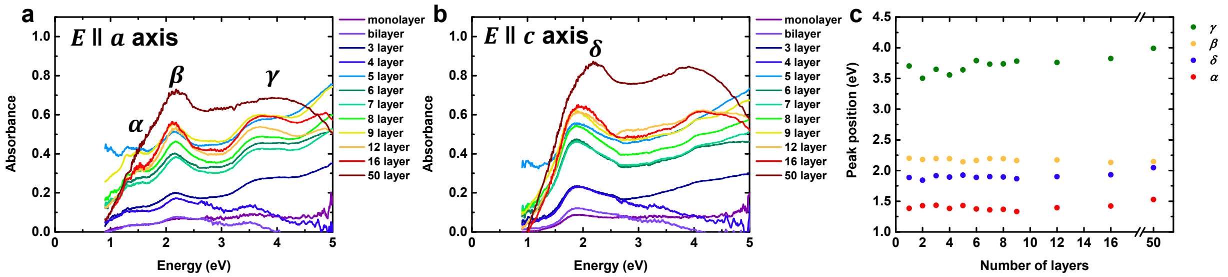


Supplementary Fig. 8: **Optical spectroscopy of ultra-thin $Ta_2NiSe_5$.** Thickness dependence of absorption spectra for 1 to 50 layers of $Ta_2NiSe_5$ at room temperature, with electric fields parallel to (**a**) the *a* axis and (**b**) the *c* axis. **c**, Peak positions of the absorption peaks as a function of thickness (number of layers), as determined by Lorentzian fitting of the spectra.

**Supplementary Note 3: Phonon modes analysis**

We measured the azimuthal-angle dependence of the phonon intensities to assign mode symmetries using Raman-tensor selection rules (Supplementary Fig. 9). In the high-temperature orthorhombic phase, the $B_{2g}$ and $A_g$ intensities in the cross polarization peak at 0° and 45°, respectively. Across all thicknesses, down to the monolayer, the polarization dependences re-

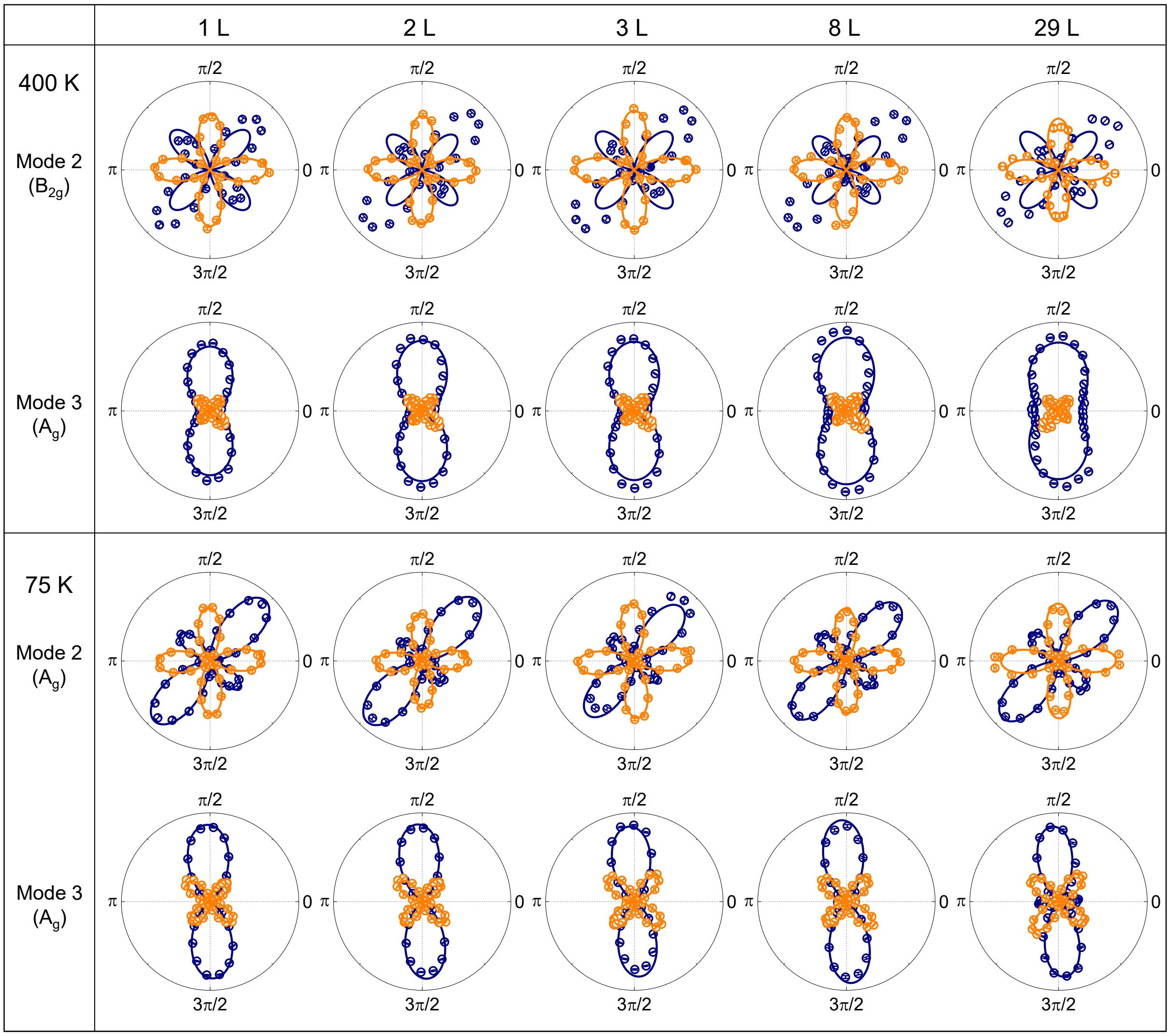


Supplementary Fig. 9: **Azimuthal angle dependence of the phonon modes.** Polarization plot of Raman intensity depending on the angle between the incident polarization and the *a*-axis of the flake, above and below the transition temperature for the ultrathin layers of $Ta_2NiSe_5$ with various thicknesses. Parallel- and cross-polarization configurations are shown in navy and orange, respectively.

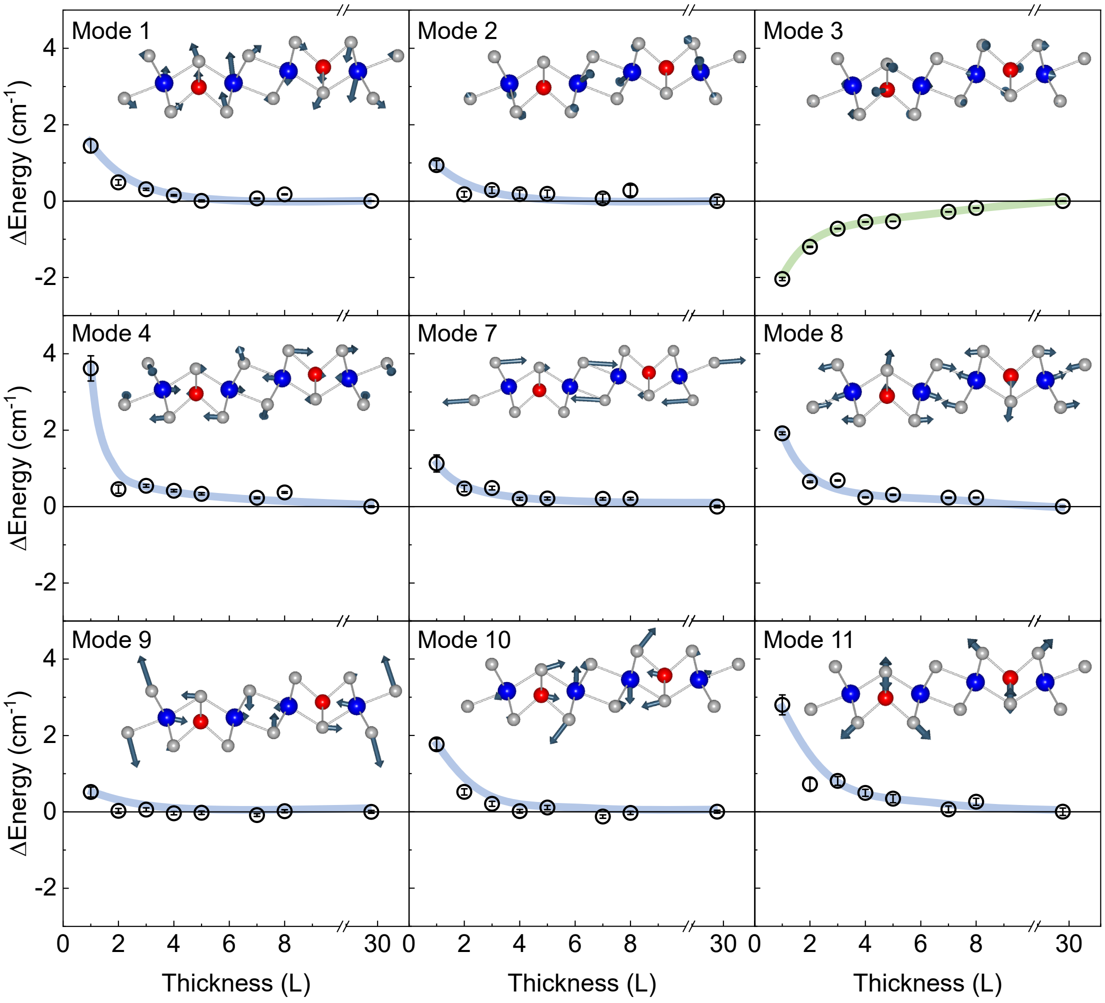


Supplementary Fig. 10: **Thickness dependence of phonon vibration frequency.** Thickness-dependent Raman frequencies taken at 75 K for each mode. The blue and green lines are the guide for the eyes. The insets are schematic illustrations of the calculated vibration patterns for each Raman-active phonon mode.

produce the bulk behavior [10], and the same agreement holds in the low-temperature phase. These results indicate that ultrathin $Ta_2NiSe_5$ preserves the intrinsic lattice symmetry and Raman selection rules of the bulk.

At 75 K, the Raman mode frequencies exhibit a systematic blue-shift with decreasing thickness for all modes except mode 3, which shows the opposite trend (a red-shift) (Supplementary Fig. 10). Similar blue-shifts of in-plane Raman modes with decreasing thickness have been reported in other two-dimensional semiconductors such as $MoS_2$ [11] and are commonly attributed to reduced dielectric screening of the long-range Coulomb interaction

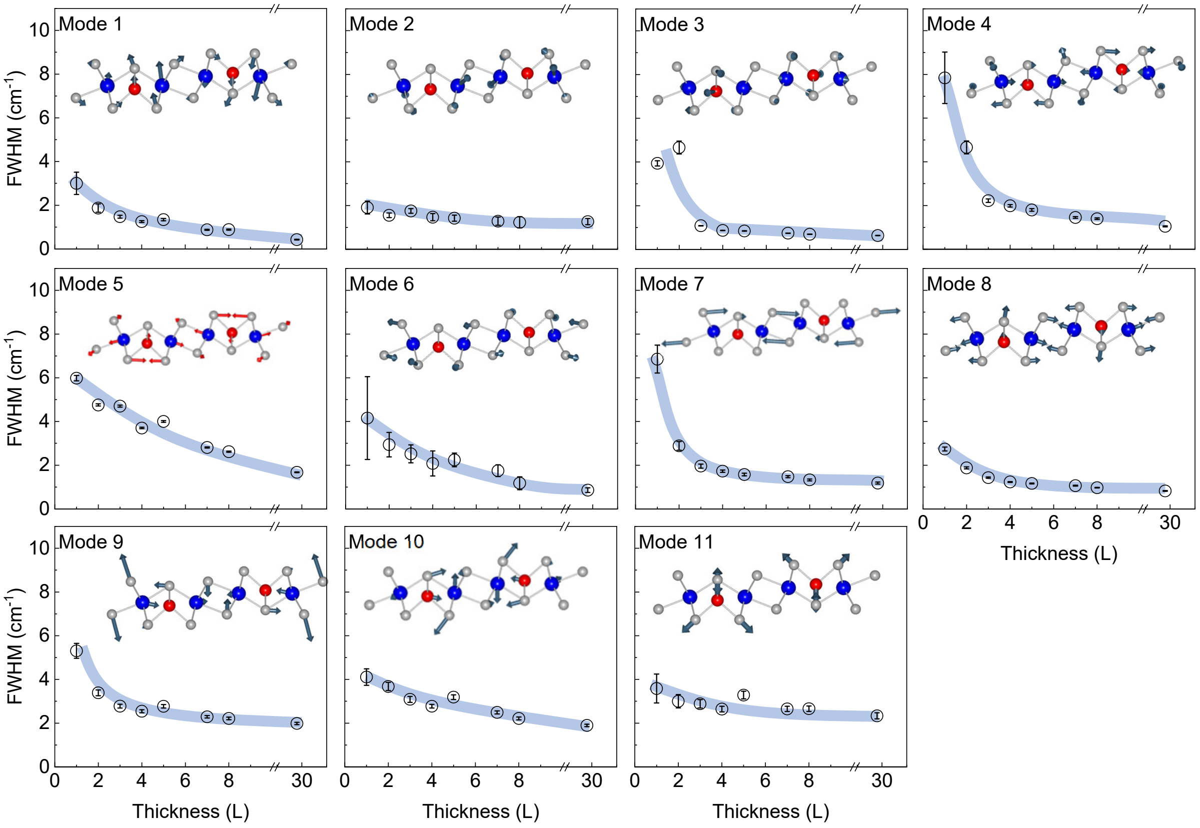


Supplementary Fig. 11: **Thickness dependence of the full-width-at-half-maximum for all the Raman modes.** The full-width-at-half-maximum (FWHM) of all the Raman modes taken at 75 K increase with decreasing thickness, resulted from the surface corrugation of the $Al_2O_3$ substrate. The blue lines are the guide for the eyes. The insets are schematic illustrations of the calculated vibration patterns for each Raman-active phonon mode.

at lower thickness [12]. Taken together, these observations indicate that ultrathin $Ta_2NiSe_5$ layers retain their intrinsic lattice structure without significant degradation during exfoliation.

As for the FWHM for Raman modes taken at 75 K, we presented the results of all eleven phonon modes. They are commonly enhanced with lowering thickness as the strain disorder effect become significant due to corrugated surface of the underlying $Al_2O_3$ layers. However, some phonon modes, such as the modes 4, 5, and 7 show more stronger thickness dependence than the other. It appears that those modes involved with dominant Se vibrational motions along the perpendicular directions of the Ta-Ni-Ta chains within the plane are affected more strongly to the corrugation in the substrate. Although detailed calculations are required for

phonon frequency shift with compressive and tensile strains along both parallel or perpendicular directions of the Ta-Ni-Ta chains, this seems related to the quasi-one-dimensional structure of $Ta_2NiSe_5$.

In Supplementary Fig. 12, we presented the temperature-dependent phonon frequency of six representative Raman modes, denoted by 2, 3, 4, 5, 8, and 9 modes, with the larger amplitude than the other modes as shown in Fig. 1 of the main text. For the samples with different thickness investigated down to monolayer, we found no signature of phonon softening behavior. Moreover, these Raman frequency below $T_c$ of $Ta_2NiSe_5$ nanoflakes exhibit the nearly same temperature dependence irrelative to their thickness. The temperature dependence of the phonon frequency is described by the phonon decay model, $\omega(T) = \omega_0 - A\left[1 + \frac{2}{e^{\hbar\omega_0/(2k_BT)}-1}\right]$, where $A$ is a positive constant determining the temperature dependence. After determining the coefficient, A from the best fit to this model for the data of the thick 29 L sample below $T_c$, we plotted the calculated $\omega(T)$ curves with the same A but adjusted $\omega_0$ for different thicknesses 1L-4L. Excellent agreement between measured and calculated $\omega(T)$ data indicate that the phonon-phonon interaction strength reflecting in the temperature dependent Raman frequencies is nearly thickness independent, in contrast clear reduction of Tc approaching to monolayer. These observations support that thickness dependence of Tc in $Ta_2NiSe_5$ nanoflakes cannot be explained by pure structural instabilities.

In the temperature-dependent phonon frequency (Supplementary Fig. 12), the frequency of Raman mode 5 decreases significantly with increasing temperature below $T_c$, which is in good agreement with previous studies [10, 13, 14]. The other modes also exhibit similar but weaker temperature dependence. Below $T_c$, all the phonon modes belong to the same $A_g$ representations, and the temperature dependent frequency of each modes reflects its different interaction strength with other phonon modes. In the phonon decay model, the effects of volume thermal expansion and anharmonic phonon-phonon interactions contribute to the temperature dependent frequency shift differently for each mode. At this stage, it is unclear why the phonon mode 5, associated with opposite vibrational motion of two neighboring Ta-Ni-Ta chains against with each other along the $c$-axis, shows stronger temperature dependence than the others. These observations are certainly very interesting and worth for further investigation, which will be done in the future work.

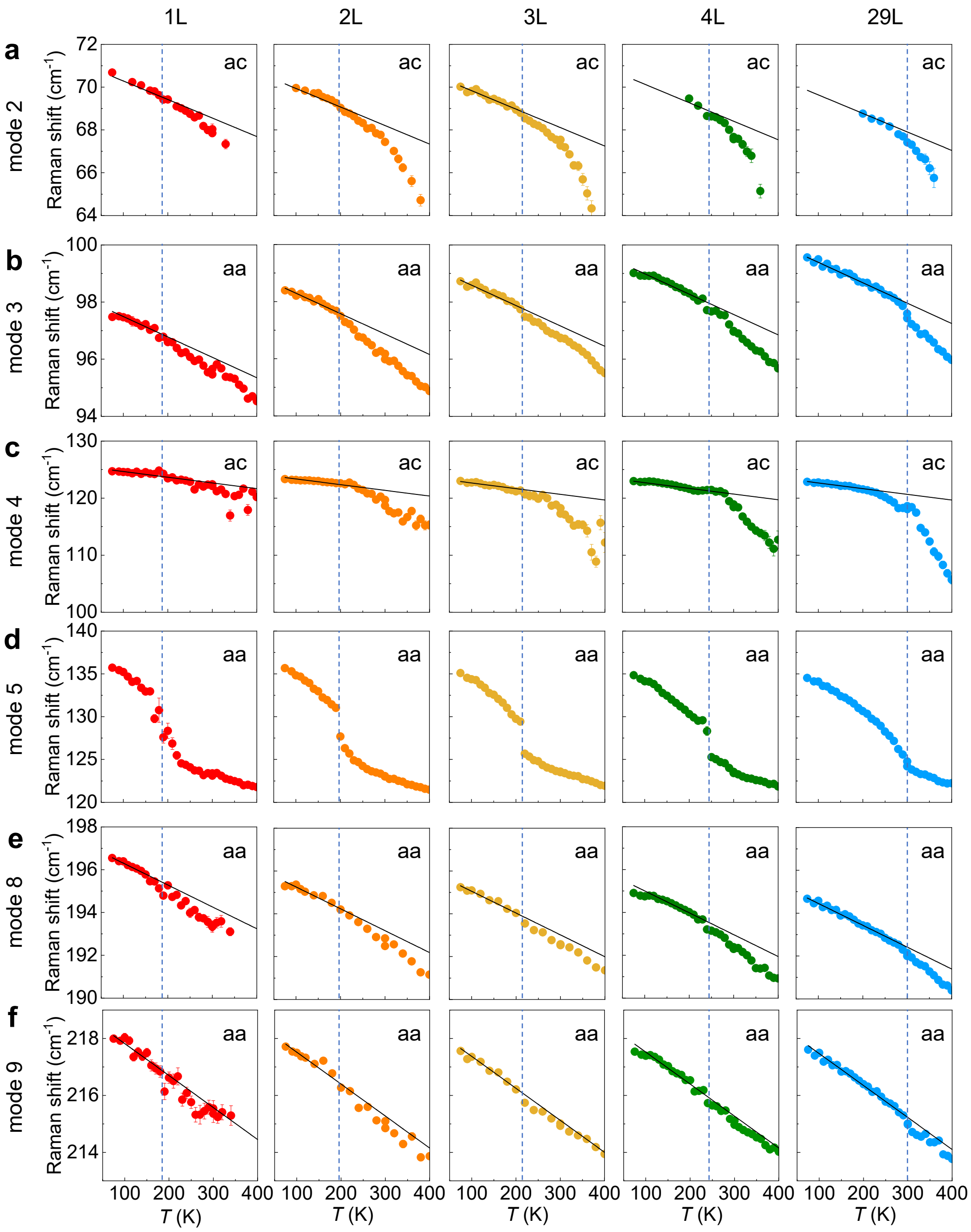


Supplementary Fig. 12: **Temperature dependent Raman frequencies of $Ta_2NiSe_5$ nanoflakes.** **a**, Raman frequency of mode 2 as a function of temperature for nanoflakes with different thicknesses of 1L, 2L, 3L, 4L and 29L. The dashed line indicates the transition temperature, $T_c$. The solid lines represent the best fit the phonon decay model for the data of 29L below $T_c$, adusted vertically for each data with different thickness. **b-f**, Similar temperature dependent Raman frequency plots for modes 2, 3, 4, 8, and 9 taken in either $ac$(**a, c**) or $aa$(**b, d-f**) polarization configurations.

## Supplementary Note 4: Phase transition

From the temperature evolution of the phonon modes, we confirm that mode 5 corresponds to $A_g$ symmetry and mode 4 corresponds to $B_{2g}$ symmetry. The blue symbols on Raman spectra in Supplementary Figs. 13a and 13b indicate the maximum intensity of the

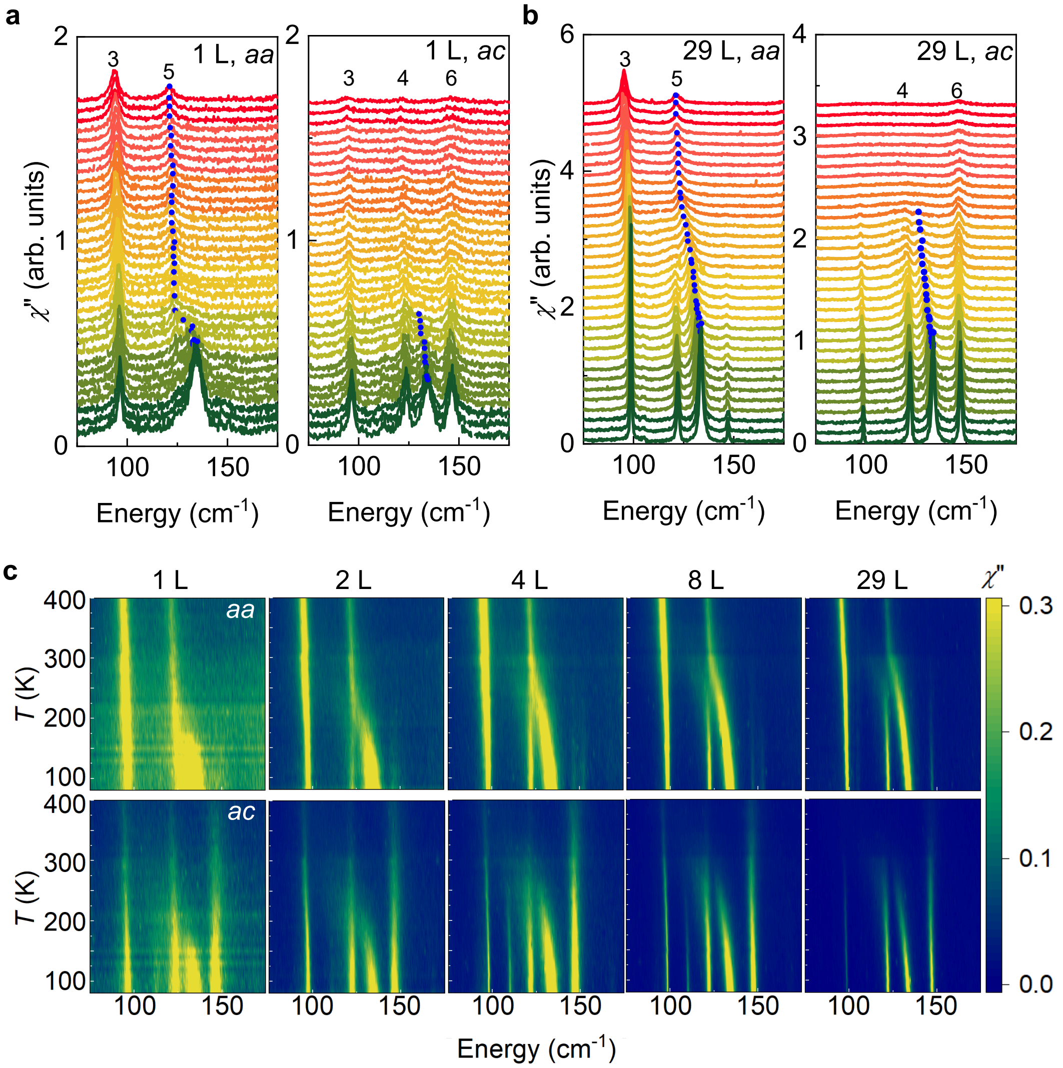


Supplementary Fig. 13: **Phonon intensity variation with temperature. a**, **b**, Raman conductivity as temperature increases from 75 K (green) to 400 K (red) measured in *aa* and *ac* configuration for monolayer and 29 layers. The numbers at the top of the graph indicate the phonon mode number and all spectra are shifted vertically for clarity. The blue points indicate the maximum intensity of the phonon mode 5. **c**, False color map of the polarized Raman spectra in the *aa* and *ac* configurations with various thickness.

phonon mode 5. In the *aa* geometry this peak persists up to 400 K, whereas in the *ac* geometry it vanishes at $T_c$. In the false color map (Supplementary Fig. 13c), this behavior is well represented for different thicknesses: at high temperature, mode 5 is present in *aa* but absent in *ac*, conversely, mode 4 remains in *ac* and disappears in *aa*. These polarization-selection trends identify that mode 5 as $A_g$ symmetry and mode 4 as $B_{2g}$ symmetry, in good agreement with the calculations in Ref. [15]. Therefore, to determine transition temperature $T_c$, we tracked mode 5 in the *ac* configuration, which exhibits the most discernible intensity across all thicknesses, by fitting the Raman spectra to the Lorentzian line shape

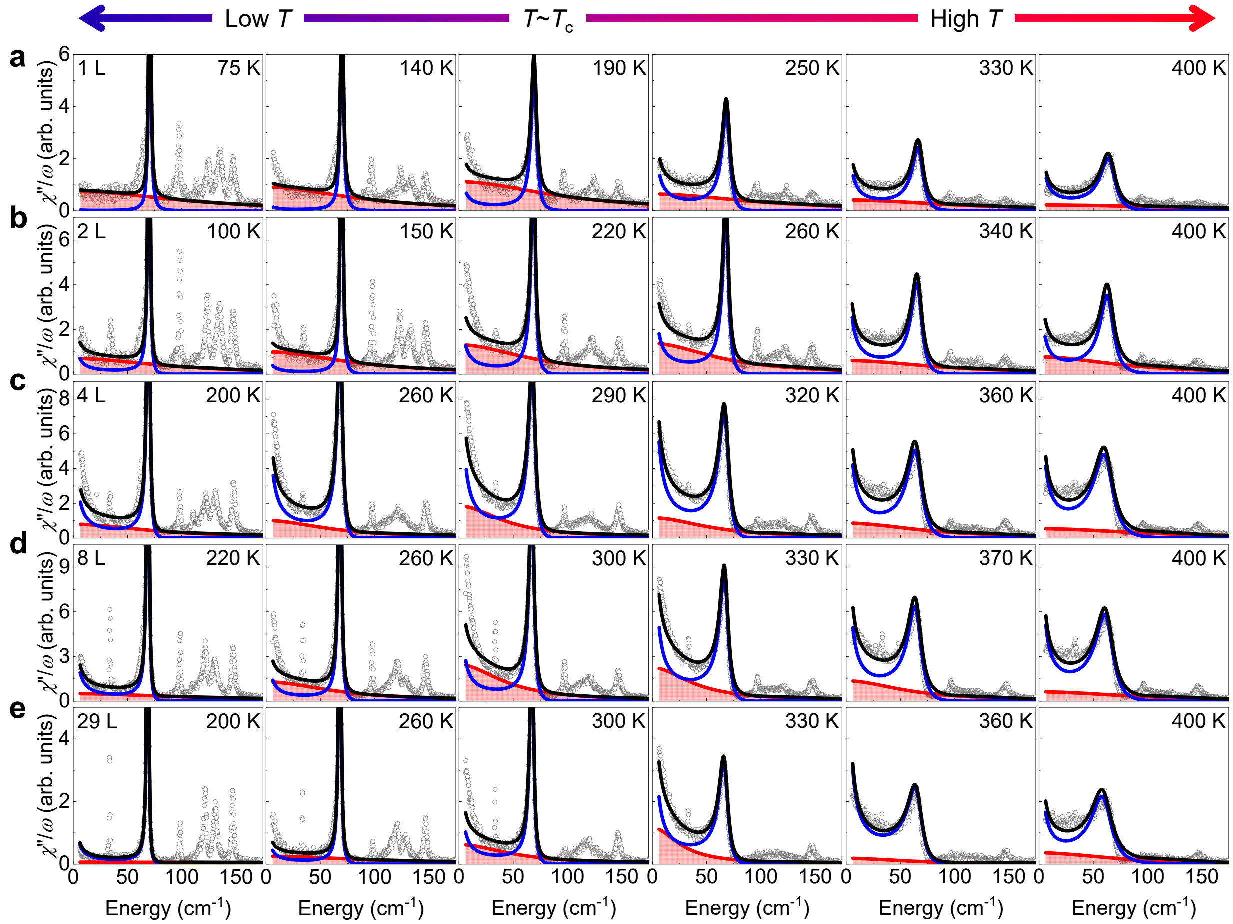


Supplementary Fig. 14: **Raman conductivity fitting of monolayer and few layer $Ta_2NiSe_5$.** Raman conductivity and the fitting results of 1 L (**a**), 2 L (**b**), 4 L (**c**), 8 L (**d**), and 29 L (**e**) at various temperature. The measured data are shown as grey dots together with the fitting results (black), which are the sum of the phononic part (blue line) and the electronic part (red line). The shaded region yields static susceptibility.

as mentioned in Ref. [16].

We fit the Raman conductivity spectra $\chi''/\omega$, taking into account two contributions of the electronic ($\chi''_{\mathrm{e}}$) and phononic ($\chi''_{\mathrm{p}}$) parts, as discussed in the main text (Supplementary Fig. 14). The measured data (gray dots) are well captured by the total fit (black line), which is the sum of the electronic (red line) and phononic (blue line) components. For all thicknesses, the electronic part (red shaded region) exhibits a pronounced critical enhancement near $T_c$ and decreases upon further heating.

The characteristic features of excitonic instability associated phase transition, substantial enhancement of low-energy Raman susceptibility near $T_c$ in the ac configuration, mode 5 disappearance at $T_c$, the Curie-Weiss-like behavior of the integrated Raman signal above $T_c$, and Fano resonance with phonon mode 2, are observed for all thicknesses as shown Supplementary Fig. 15. The broad continuum of electronic excitations at low frequency emerges with increasing temperature, reaches its maximum intensity at $\sim T_c$, and then diminishes above $T_c$ (Supplementary Fig. 15a). By tracking the integrated area of mode 5, as fitting the Raman spectra with a Lorentzian line shape, we determined $T_c$, which is lowered as thickness is reduced from 29 L to monolayer (Supplementary Fig. 15b). Both the low energy $\chi''/\omega$ and the static susceptibility $\chi$, obtained by integrating the electronic Raman conductivity $\chi''_e/\omega$ over the whole energy range, exhibit a clear maximum at $\sim T_c$ estimated from the temperature-dependent intensity of phonon mode 5 (Supplementary Fig. 15c). The best fit to the Fano resonance model consistently yields the asymmetry parameter $q$ reduced with lowering thickness (Supplementary Fig. 15d), indicating weak suppression of exciton-phonon coupling. Clearly, the signatures of the phase transition persist even approaching the monolayer limit, albeit systematically suppressed.

We prepared additional monolayer flakes using the same single crystal. For each preparation we employed the same procedure and conditions for $Al_2O_3$ layer deposition such as a deposition rate of 0.1 Å/s and ultra-high vacuum level of $\sim 10^{-7}$ Torr. The resulting three monolayer flakes were investigated with Raman spectroscopy and compared with the results presented in the main text. As shown in Supplementary Fig. 16, these additional samples exhibit the qualitatively same Raman spectra and the similar temperature dependence. The transition temperature was estimated from the normalized integrated area of the phonon mode 5 and broad maximum of the low-energy Raman susceptibility $\chi''/\omega$ as a function of temperature. The estimated transition temperatures are $\sim$ 190 K – 215 K, in reasonable

agreement with each other and far less than the bulk value $T_c \sim 335$ K.

We conducted Raman spectroscopy measurements on $Ta_2NiS_5$, the sulfur analogue of $Ta_2NiSe_5$. It is well established that $Ta_2NiS_5$ possesses a direct band gap larger than the excitonic binding energy, which significantly suppresses excitonic instabilities. None of the main Raman features indicative of excitonic instabilities observed in $Ta_2NiSe_5$, such as the diverging signatures of low-energy excitations and Fano-shaped phonon modes, were detected in bulk $Ta_2NiS_5$ [17]. The same conclusion holds for nanoflakes of $Ta_2NiS_5$, which were obtained using the same method as in this work. In contrast to $Ta_2NiSe_5$, we did not observe any clear anomalies in the temperature-dependent frequency or full-width-half-maximum of the phonon modes, nor any significant diverging behavior in low-energy excitations down to 77 K. The absence of phase transition signatures in $Ta_2NiS_5$ indicates that it remains well within the insulating regime, where the electronic band gap exceeds the excitonic binding energy, significantly suppressing excitonic instabilities. As discussed in Supplementary Fig. 6, the electronic structure of $Ta_2NiSe_5$ remains largely unchanged with decreasing thickness, and a similar behavior is likely for $Ta_2NiS_5$.

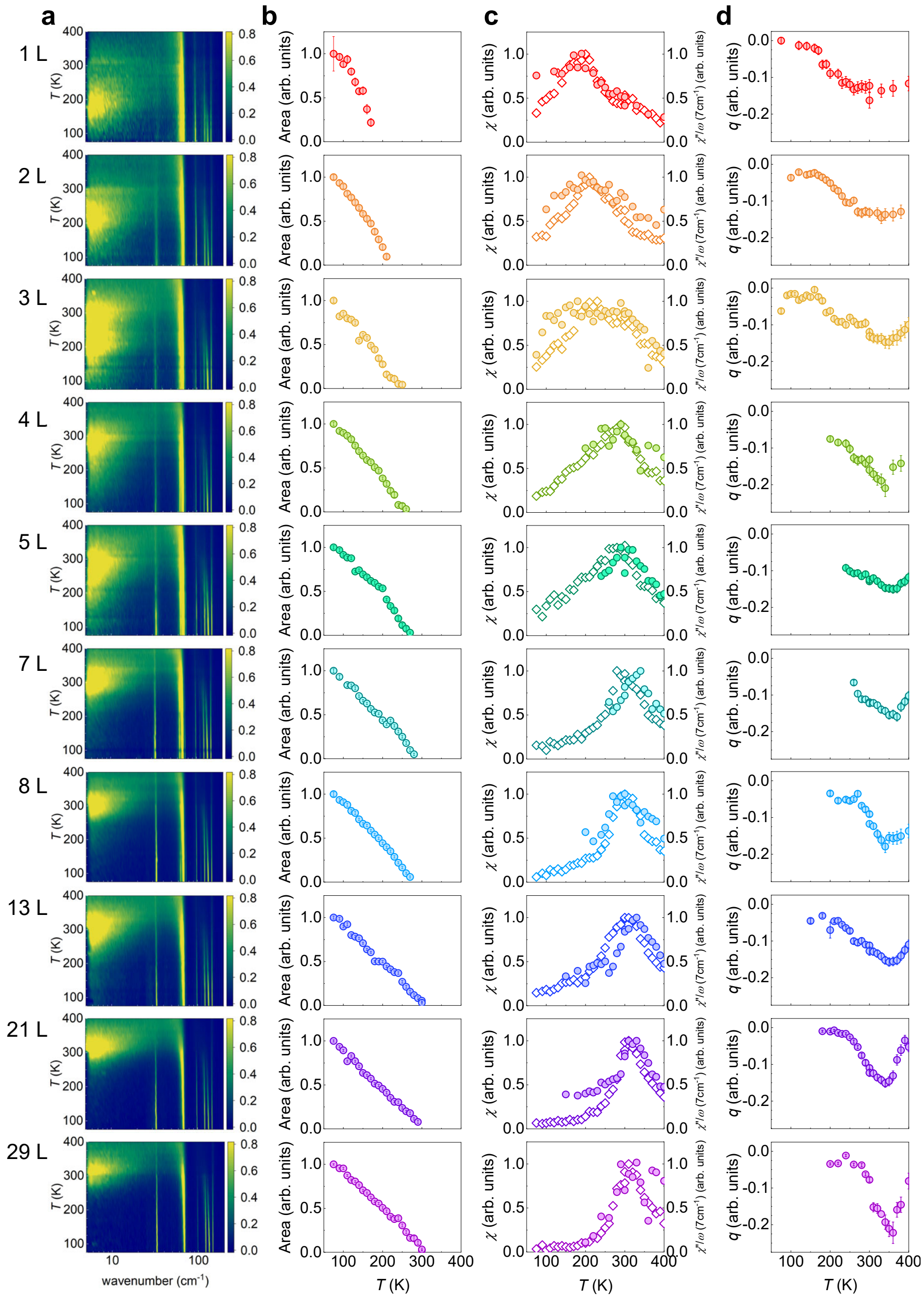


Supplementary Fig. 15: **Raman spectroscopy results for $Ta_2NiSe_5$ with different thickness.** **a**, False color map showing the normalized Raman conductivity in the *ac* configuration from 75 K to 400 K for eleven samples with different thickness from 1L to 29L. **b**, Normalized integrated area of the phonon mode 5 as a function of temperature. **c**, Normalized Raman conductivity at 7 $cm^{-1}$ (diamond) and normalized static susceptibility (circle) as a function of temperature. **d**, Asymmetric parameter $q$ obtained from the Fano fitting of the phonon mode 2.

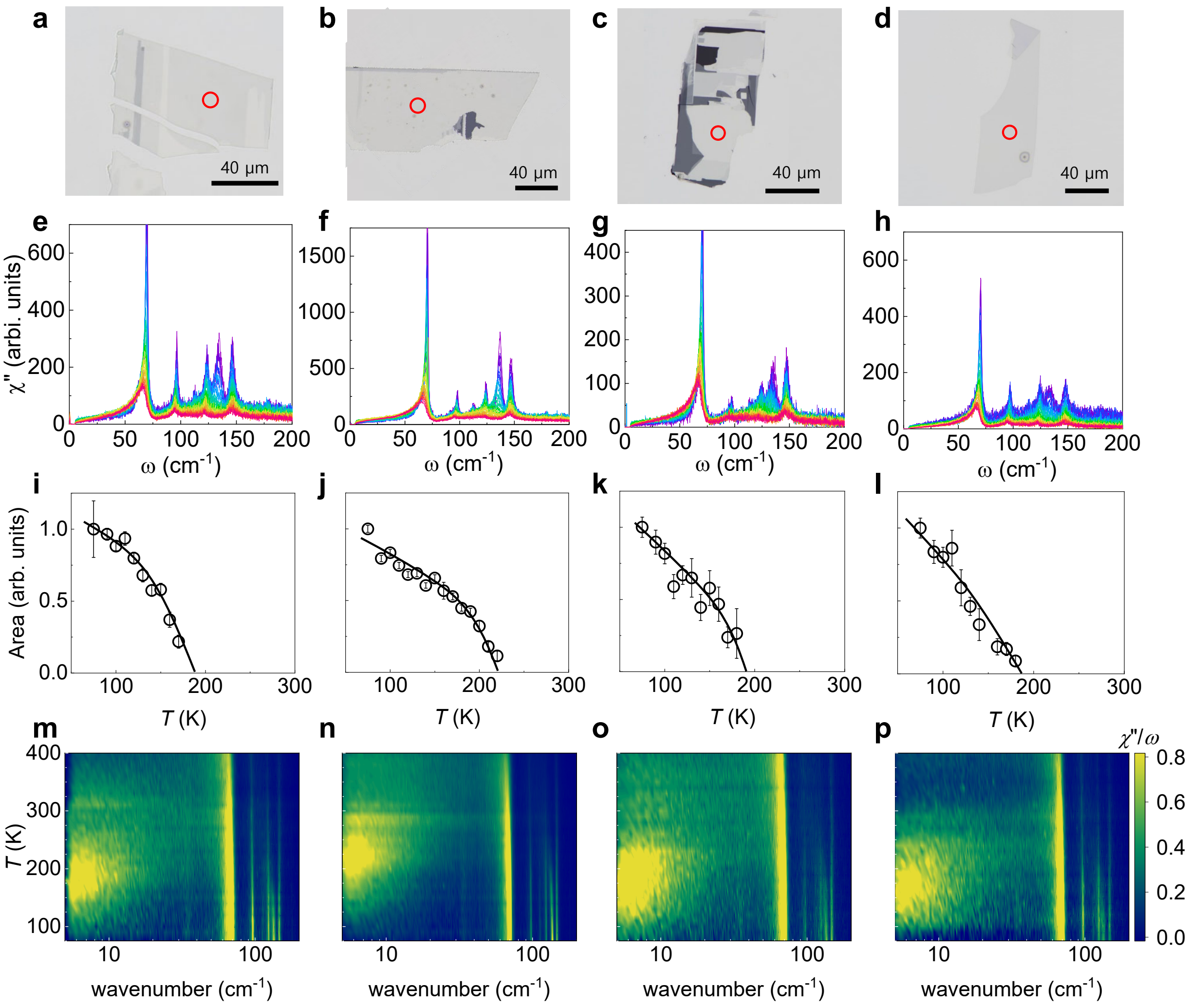


Supplementary Fig. 16: **Raman spectroscopy results for different $Ta_2NiSe_5$ monolayer flakes. a-d**, Optical images of four $Ta_2NiSe_5$ monolayer flakes where the Raman spectra were taken at the position indicated by red circles. **e-h**, Corresponding Raman spectra at different temperatures. **i-l**, Temperature dependence of the spectral area for Raman mode 5. The solid lines are the guide-to-the-eyes. **m-p**, Contour plots for Raman susceptibility $\chi''/\omega$ as a function of wavenumber and temperature.

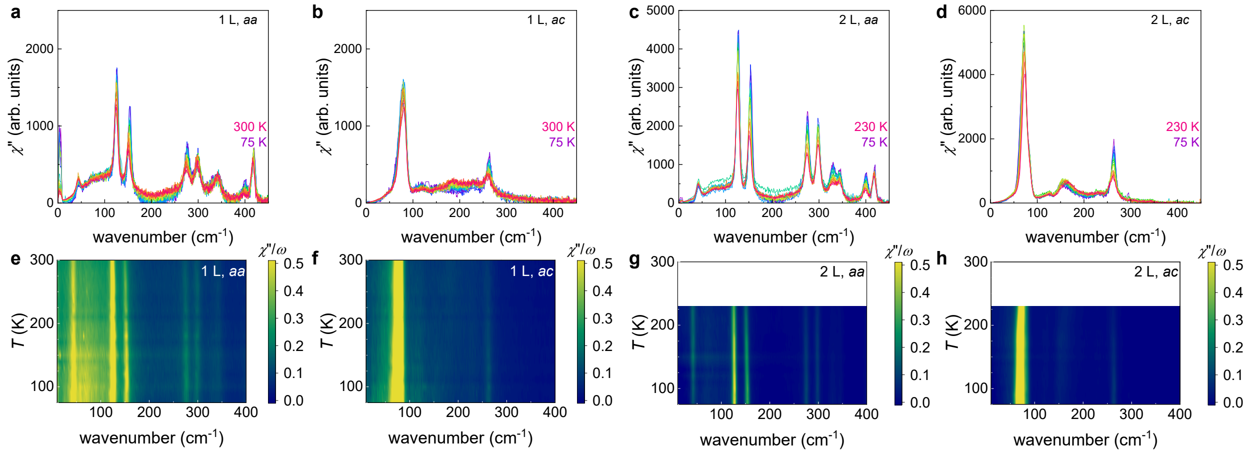


Supplementary Fig. 17: **Raman spectroscopy on monolayer and bilayer $Ta_2NiS_5$. a-d**, Raman spectra $\chi''$ in the $aa$(**a, c**) and $ac$(**b, d**) configurations for monolayer (**a, b**) and bilayer (**c, d**) $Ta_2NiS_5$. **e-f**, Corresponding false color map of Raman conductivity $\chi''/\omega$ for monolayer (**e, f**) and bilayer (**g, h**) $Ta_2NiS_5$.

**Supplementary Note 5: Temperature dependent resistivity without semimetal-insulator transition**

We emphasize that the observed features in our Raman spectroscopy data cannot be explained by a purely structural origin. Within the framework of a purely structural phase transition, phenomena such as the Curie-Weiss behavior of low-energy excitations and the significant enhancement of Fano resonance in specific phonon modes above $T_c$ are attributed to the presence of conducting carriers due to an insulator-to-semimetal transition. This conjecture is further supported by anomalies in the activation gap extracted from temperature-dependent resistivity measurements in bulk $Ta_2NiSe_5$. In contrast, our experiments on monolayer $Ta_2NiSe_5$ show a temperature-dependent resistivity, shown in Supplementary Fig. 18, that increases monotonically with no anomalies observed as the temperature is lowered across $T_c$, indicating negligible changes in conducting carrier density. Despite this, both the low-energy excitations and Fano resonance behavior in our data exhibit significant changes across $T_c$. (Figs. 2f, 3c, and 3d). These observations cannot be explained by a structural transition but are consistent with the critical behavior of excitonic instability at $T_c$.

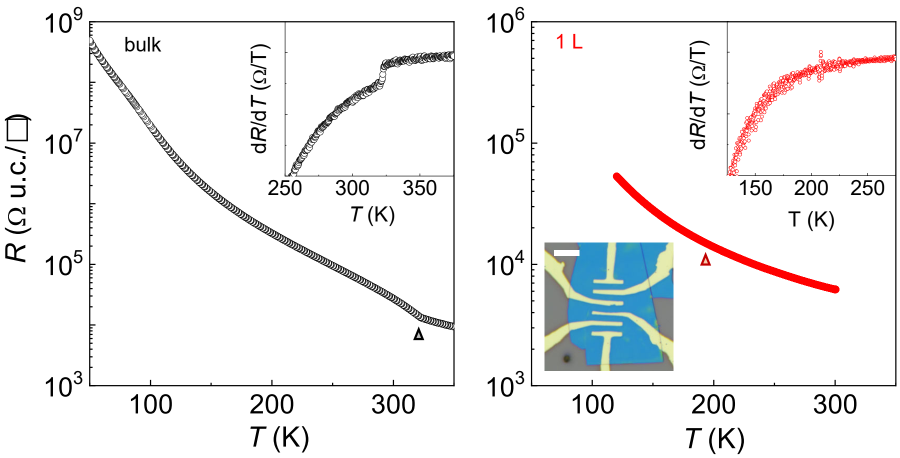


Supplementary Fig. 18: **Sheet resistance of bulk and monolayer $Ta_2NiSe_5$. a**, **b**, temperature dependent sheet resistance $R(T)$ curves of bulk (**a**) and monolayer (**b**). The insets show the temperature derivatives of $R(T)$. The transition temperature extracted from Raman spectroscopy is indicated by upper triangle for comparison. The optical image is the monolayer sample with electrodes (scale bar : 10 $\mu$m) is shown.

[1] Y. Deng, Y. Yu, Y. Song, J. Zhang, N. Z. Wang, Z. Sun, Y. Yi, Y. Z. Wu, S. Wu, J. Zhu, J. Wang, X. H. Chen, Y. Zhang, *Nature* **2018**, *563*, 7729 94.

[2] S. Y. Kim, Y. Kim, C.-J. Kang, E.-S. An, H. K. Kim, M. J. Eom, M. Lee, C. Park, T.-H. Kim, H. C. Choi, B. I. Min, J. S. Kim, *ACS Nano* **2016**, *10*, 9 8888.

[3] W. Hayes, R. Loudon, *John Wiley and Sons, New York* **1978**.

[4] T. H. Kauffmann, N. Kokanyan, M. D. Fontana, *Journal of Raman Spectroscopy* **2019**, *50*, 3 418.

[5] J. Lee, C.-J. Kang, M. J. Eom, J. S. Kim, B. I. Min, H. W. Yeom, *Phys. Rev. B* **2019**, *99*, 7 075408.

[6] K. Seki, Y. Wakisaka, T. Kaneko, T. Toriyama, T. Konishi, T. Sudayama, N. L. Saini, M. Arita, H. Namatame, M. Taniguchi, N. Katayama, M. Nohara, H. Takagi, T. Mizokawa, Y. Ohta, *Phys. Rev. B* **2014**, *90*, 15 155116.

[7] S. Lee, K.-H. Jin, H. Jung, K. Fukutani, J. Lee, C. I. Kwon, J. S. Kim, J. Kim, H. W. Yeom, *ACS nano* **2024**, *18*, 36 24784.

[8] K. Katsumi, A. Alekhin, S.-M. Souliou, M. Merz, A.-A. Haghighirad, M. Le Tacon, S. Houver, M. Cazayous, A. Sacuto, Y. Gallais, *Physical Review Letters* **2023**, *130*, 10 106904.

[9] K. Sugimoto, S. Nishimoto, T. Kaneko, Y. Ohta, *Phys. Rev. Lett.* **2018**, *120*, 24 247602.

[10] K. Kim, H. Kim, J. Kim, C. Kwon, J. S. Kim, B. J. Kim, *Nature Communications* **2021**, *12*, 1 1969.

[11] C. Lee, H. Yan, L. E. Brus, T. F. Heinz, J. Hone, S. Ryu, *ACS nano* **2010**, *4*, 5 2695.

[12] A. Molina-Sánchez, L. Wirtz, *Phys. Rev. B* **2011**, *84*, 15 155413.

[13] D. Werdehausen, T. Takayama, M. Höppner, G. Albrecht, A. W. Rost, Y. Lu, D. Manske, H. Takagi, S. Kaiser, *Science Advances* **2018**, *4*, 3 eaap8652.

[14] M.-J. Kim, A. Schulz, T. Takayama, M. Isobe, H. Takagi, S. Kaiser, *Physical Review Research* **2020**, *2*, 4 042039.

[15] M. Ye, P. A. Volkov, H. Lohani, I. Feldman, M. Kim, A. Kanigel, G. Blumberg, *Phys. Rev. B* **2021**, *104*, 4 045102.

[16] S. Mor, M. Herzog, J. Noack, N. Katayama, M. Nohara, H. Takagi, A. Trunschke, T. Mizokawa, C. Monney, J. Stähler, *Physical Review B* **2018**, *97*, 11 115154.

[17] P. A. Volkov, M. Ye, H. Lohani, I. Feldman, A. Kanigel, G. Blumberg, *Phys. Rev. B* **2021**, *104*, 24 L241103.
